# CCDC58 is a potential biomarker for diagnosis, prognosis, immunity, and genomic heterogeneity in pan-cancer

**DOI:** 10.1038/s41598-024-59154-9

**Published:** 2024-04-13

**Authors:** Kai Yang, Yan Ma, Weigang Chen, Lu Liu, Zelong Yang, Chaokui He, Nanbei Zheng, Xinyu Liu, Xin Cheng, Junbo Song, Yong Chen, Hongyu Qiao, Ruohan Zhang

**Affiliations:** 1 Department of Hepatobiliary Surgery, Xi Jing Hospital, Air Force Medical University, Xi’an, 710032 China; 2 Department of Gynecology and Obstetrics, Xi Jing Hospital, Air Force Medical University, Xi’an, 710032 China; 3 College of Life Sciences, Northwest University, Xi’an, 710000 China; 4 Department of Oncology, The First Affiliated Hospital of Shanxi Medical University, Taiyuan, 030000 China; 5 Department of General Surgery, The First Affiliated Hospital of Jiamusi University, Jiamusi, 154002 China; 6 Department of Pediatrics, Xi Jing Hospital, Air Force Medical University, Xi’an, 710032 China

**Keywords:** CCDC58, Pan-cancer, Biomarker, Immune infiltration, LIHC, Tumour biomarkers, Protein function predictions

## Abstract

Coiled-coil domain-containing 58 (CCDC58) is a member of the CCDC protein family. Similar to other members, CCDC58 exhibits potential tumorigenic roles in a variety of malignancies. However, there is no systematic and comprehensive pan-cancer analysis to investigate the diagnosis, prognosis, immune infiltration, and other related functions of CCDC58. We used several online websites and databases, such as TCGA, GTEx, UALCAN, HPA, CancerSEA, BioGRID, GEPIA 2.0, TIMER 2.0, and TISIDB, to extract CCDC58 expression data and clinical data of patients in pan-cancer. Then, the relationship between CCDC58 expression and diagnosis, prognosis, genetic alterations, DNA methylation, genomic heterogeneity, and immune infiltration level were determined. In addition, the biological function of CCDC58 in liver hepatocellular carcinoma (LIHC) was investigated. Pan-cancer analysis results showed that CCDC58 was differentially expressed in most tumors and showed excellent performance in diagnosis and prediction of prognosis. The expression of CCDC58 was highly correlated with genetic alterations, DNA methylation, and genomic heterogeneity in some tumors. In addition, the correlation analysis of CCDC58 with the level of immune infiltration and immune checkpoint marker genes indicated that CCDC58 might affect the composition of the tumor immune microenvironment. Enrichment analysis showed that CCDC58-related genes were mainly linked to mitosis, chromosome, and cell cycle. Finally, biological function experiments demonstrated that CCDC58 plays an important role in tumor cell proliferation and migration. CCDC58 was first identified as a pan-cancer biomarker. It may be used as a potential therapeutic target to improve the prognosis of patients in the future.

## Introduction

Coiled-coil domain was a highly conserved supercoiled protein motif with several alpha-helices, usually two to six. It was estimated that about 10% of proteins contain coiled-coil domains^1,2^. Coiled-coil domain-containing (CCDC) proteins were expressed in many tissues, and involved in many important physiological functions^3^. At present, it had been reported that CCDC proteins took key roles in intracellular signal transduction, genetic signal transcription, cell cycle regulation, and other functions^4^. In recent years, more and more scholars paid attention to the expression and effect of CCDC proteins in malignant tumors. For example, Zhen Liu et al.^5^ pointed out that CCDC19 might play a tumor suppressor role by inhibiting the proliferation, invasion, and migration of nasopharyngeal carcinoma cells. It had been suggested that CCDC67 was a tumor suppressor gene that was silenced by promoter CpG methylation in gastric cancer, and it might be involved in cell signaling and migration related to tumorigenesis^6^. With literature, circ-CCDC66 was up-regulated in renal cancer cell lines and could enhance the enrichment of tumor stem cells^7^. In addition, CCDC85B promotes the proliferation and invasion of non-small cell lung cancer cells by activating the AKT/GSK3β/β-catenin oncogenic signaling pathway^8^. It had been confirmed that overexpression of the CCDC34 protein in esophageal squamous cell carcinoma was associated with tumor progression, angiogenesis, and poor survival^9^. In summary, studies had confirmed that some CCDC family proteins were involved in the occurrence and development of tumors.

The CCDC58 gene, also known as Mitochondrial Matrix Import Factor 23 (MIX23), was located on chromosome 3q21.1. It encodes CCDC58 protein, including 144 amino acids, with a molecular weight of 16620 Da. CCDC58 protein belonged to intracellular proteins located in mitochondria^10^. Previous studies indicated that the knockdown of CCDC58 could enhance Ca^2+^ retention and bioenergetics in mitochondria^11^. Other scholars found that CCDC58 was significantly up-regulated when mitochondrial input was interfered with, suggesting that CCDC58 might be a stabilizer or regulator of the mitochondrial protein input mechanism^12^. In triple-negative breast cancer, a miR-432-p/CCDC58 regulatory axis influenced tumor proliferation, migration, and invasion phenotypes^13^. CCDC58 had also been identified as a gene associated with tumor progression in endometrial cancer and ovarian cancer^14,15^. Among the differentially expressed genes between diabetic patients and normal controls, CCDC58 ranked in the top ten, suggesting that CCDC58 might be related to the occurrence and development of diabetes^16^.

Given the lack of studies on CCDC58 and its potential role in tumorigenesis, we performed a pan-cancer analysis of CCDC58. Based on several reliable databases and combined with statistical analysis, we showed diverse results of CCDC58 expression, diagnostic and prognostic value, genomic heterogeneity, immune infiltration analysis, enrichment analysis, and so on. Finally, We explored the role of CCDC58 in progression of liver cancer cells, analyzed its correlation with clinical indicators, and established a prognostic nomogram in liver hepatocellular carcinoma (LIHC). It was concluded that CCDC58 was a reliable pan-cancer biomarker, and it had good diagnostic and prognostic value in a variety of cancers. The analysis and experimental results of this study provided a direction for tumor treatment.

## Materials and methods

### CCDC58 expression analysis

We downloaded RNA-seq data of pan-cancer in transcripts per million (TPM) format of The Cancer Genome Atlas (TCGA) and The Genotype-Tissue Expression (GTEx) Project uniformly processed by Toil^17^ process from UCSC XENA(https://xenabrowser.net/datapages/)^18^. Types of cancer included adrenocortical carcinoma (ACC), bladder urothelial carcinoma (BLCA), breast invasive carcinoma (BRCA), cervical squamous cell carcinoma and endocervical adenocarcinoma (CESC), cholangio carcinoma (CHOL), colon adenocarcinoma (COAD), lymphoid neoplasm diffuse large B-cell lymphoma (DLBC), esophageal carcinoma (ESCA), glioblastoma multiforme (GBM), head and neck squamous cell carcinoma (HNSC), kidney chromophobe (KICH), kidney renal clear cell carcinoma (KIRC), kidney renal papillary cell carcinoma (KIRP), acute myeloid leukemia (LAML), brain lower grade glioma (LGG), LIHC, lung adenocarcinoma (LUAD), lung squamous cell carcinoma (LUSC), mesothelioma (MESO), ovarian serous cystadenocarcinoma (OV), pancreatic adenocarcinoma (PAAD), pheochromocytoma and paraganglioma (PCPG), prostate adenocarcinoma (PRAD), rectum adenocarcinoma (READ), sarcoma (SARC), skin cutaneous melanoma (SKCM), stomach adenocarcinoma (STAD), testicular germ cell tumors (TGCT), thyroid carcinoma (THCA), thymoma (THYM), uterine corpus endometrial carcinoma (UCEC), uterine carcinosarcoma (UCS) and uveal melanoma (UVM). The log_2_(TPM + 1) data was applied for the log scale. R “stats” and “car” packages were used for statistical analysis according to the data characteristics, and R “ggplot2” package was used to visualize the statistical results^19–21^. The results of the level of protein expression and the relationship between CCDC58 expression and individual cancer stage were obtained from the Clinical Proteomic Tumor Analysis Consortium (CPTAC) of the University of ALabama at Birmingham CANcer data analysis Portal (UALCAN, http://ualcan.path.uab.edu/analysis-prot.html)^22^. The Human Protein Atlas (HPA, https://www.proteinatlas.org/)^23^ was further employed to show the expression of CCDC58 in tumor and normal tissue.

### Survival and ROC curve analysis

The Kaplan–Meier (K–M) survival curves were used to display the correlation between the expression of CCDC58 and Overall Survival (OS) and Disease-Specific Survival (DSS) of cancers using TCGA datasets. Patients were divided into 0–50% and 50–100% groups according to the expression levels of CCDC58. Samples with no clinical information were removed^24^. Log-rank *P* value and 95% confidence intervals (CI) were examined. R “survival” package was used for fitting survival regression, and the results were visualized using R “survminer” and “ggplot2” package^25^. Based on Receiver Operating Characteristic (ROC) curve, the Area Under Curve (AUC) was often used to evaluate the diagnostic effect of variables in predicting outcomes. R “pROC” package and “timeROC” package were used for ROC analysis of the data from TCGA and GTEx, and the results were visualized with R “ggplot2” package^26,27^.

### Critical clinical characteristics analysis

R “stats” package was used to analyze the correlation between CCDC58 expression and clinical characteristics. Statistically significant results were presented in the form of a three-line table.

### Genetic alteration and DNA methylation analysis

We obtained the Genetic Alteration of the CCDC58 gene in cBioPortal (https://www.cbioportal.org/))^28,29^, as well as Structural variant data, Mutation data, and Copy Number Alteration (CNA) data. In addition, R “ggplot2” package was used to visualize RNA-seq data and Illumina human methylation 450 data of CCDC58 from TCGA.

### Genomic heterogeneity analysis

We obtained the scores of 8 tumor genomic heterogeneity indicators from UCSC and TCGA. And based on R “maftools” package, we analyzed the Pearson correlation between them and the expression of CCDC58, including tumor mutation burden (TMB), mutant-allele tumor heterogeneity (MATH), microsatellite instability (MSI), neoantigen (NEO), PURITY, PLOIDY, homologous recombination deficiency (HRD), loss of heterozygosity (LOH)^30^. TMB was highly correlated with the efficacy of immune checkpoint inhibitors and could predict the efficacy of immunotherapy in tumor patients to a certain extent^31^. MATH was able to represent a bias in the distribution of mutation annotation format (MAF) values at tumor-specific mutation sites, which were positively associated with tumor heterogeneity^32^. MSI was the spontaneous loss or acquisition of nucleotides in repetitive DNA, associated with defects in DNA mismatch repair, and was a diagnostic phenotype for a variety of malignancies^33^. NEO were tumor-specific antigens derived from somatic cell mutations and were considered important targets for tumor immunotherapy^34,35^. Tumor tissue contained tumor cells, immune cells, stromal cells, stromal cells, and other cells, which jointly affected the development of the tumor. Therefore, tumor PURITY was significantly correlated with clinical characteristics, genomic expression, and biological characteristics of tumor patients^35,36^. PLOIDY was considered a hallmark of cancer and was closely related to chromosome instability in cancer development^35,37^. Homologous recombination was a high-precision DNA repair mechanism, so HRD was a key indicator of multiple tumor treatment options and prognosis^35,38^. LOH represented chromosomal events that could cause the loss of entire genes and nearby chromosomal regions^35,39^. Finally, the correlation analysis results of the above 8 indicators were displayed in the form of radar charts.

### Immune infiltration analysis

Based on single sample gene set enrichment analysis (ssGSEA) of R “GSVA” package^40^, Spearman correlation analysis was conducted between CCDC58 and 24 immune cells markers of immune infiltration matrix data from TCGA datasets. 24 immune cells included activated DC (aDC), B cells, CD8 T cells, Cytotoxic cells, DC, Eosinophils, immature DC (iDC), Macrophages, Mast cells, Neutrophils, NK CD56bright cells, NK CD56dim cells, NK cells, Plasmacytoid DC (pDC), T cells, T helper cells, T central memory (Tcm), T effector memory (Tem), T follicular helper (TFH), T gamma delta (Tgd), Th1 cells, Th17 cells, Th2 cells, TReg^41^. Combined with previous reports^35^, we also extracted the expression data of two types of immune checkpoint marker genes (inhibitory 24 and stimulatory 36) and analyzed their correlation with CCDC58 in pan-cancer. Finally, R “ggplot2” package was used for visualization.

### Single-cell sequencing

In CancerSEA (http://biocc.hrbmu.edu.cn/CancerSEA/home.jsp)^42^, the relevance of CCDC58 across 14 functional states in distinct cancers and functional relevance in distinct cell groups was obtained. T-SNE describes the distribution of cells, and the color of the dots represents the level of CCDC58 expression.

### Related genes enrichment analysis

BioGRID (https://thebiogrid.org/)^43^ was a biomedical interactive repository whose data was compiled through comprehensive administrative effort. The protein interaction network of CCDC58 can be obtained with it. In addition, Gene Expression Profiling Interactive Analysis (GEPIA 2.0, http://gepia2.cancer-pku.cn/#index)^44^ web server was a high-quality resource for gene expression analysis based on tumor and normal samples from TCGA and GTEx databases. Tumor Immune Estimation Resource (TIMER 2.0, http://timer.cistrome.org/)^45^ was a comprehensive resource for the systematic analysis of immune infiltration in different cancer types. In all tumor and normal tissue samples, the top 100 genes highly related to CCDC58 were obtained from GEPIA 2.0. We carried out a correlation analysis between the top 10 genes and CCDC58 in GEPIA 2.0 and TIMER 2.0, conducted Gene ontology (GO) and Kyoto encyclopedia of genes and genome (KEGG) enrichment analysis on the top 100 genes, based on R “clusterProfiler” package, to investigate the key phenotypes and signal pathways that CCDC58 might affect in pan-cancer^46^. Finally, R “ggplot2”, “igraph” and “ggraph” packages were used for visualization^47,48^.

### Further analysis of CCDC58 expression in LIHC

TISIDB (http://cis.hku.hk/TISIDB/index.php)^49^ was an integrated repository portal for tumor-immune system interactions. Based on CCDC58 expression, we obtained immune subtypes and molecular subtypes of LIHC in TISIDB. In addition, we analyzed correlations between CCDC58 expression and clinical variables in LIHC, GBM/LGG, SARC, and UCEC from TCGA, visualizing the results with boxplots and K–M survival curves. Based on R “survival” package, Cox regression analysis was performed for clinical variables including CCDC58, and variables with *P* < 0.1 were included in the multivariate analysis. According to the variables with *P* < 0.05 in the multivariate Cox regression analysis, R “rms” package was used to construct and visualize the OS prognosis nomogram. Finally, calibration curves were drawn and prognostic decision curve analysis (DCA)^50^ was performed.

### Cell lines and transfection

The human liver cancer cell line (MHCC97-H) was purchased from Zhong Qiao Xin Zhou Biotechnology Co., Ltd (Shanghai, China). Human liver cancer cell lines (Huh-7, HCCLM3, and Hep 3B) and human liver cell line (WRL-68) were purchased from Haixing Biological Technology Co., Ltd (Suzhou, China). The human liver cancer cell line (Hep G2) was purchased from Procell Life Science&Technology Co., Ltd (Wuhan, China). All cell lines were identified correctly by STR analysis. MHCC97-H, Huh-7, HCCLM3, and WRL-68 were cultured in Dulbecco’s Modified Eagle Medium (DMEM, Gibco, USA) + 10%Fetal Bovine Serum (FBS, Gibco, USA) + 1%Antibiotics (Penicillin streptomycin, Gibco, USA). Hep G2 and Hep 3B were cultured in Minimum Essential Medium (MEM, Gibco, USA) + 10%Fetal Bovine Serum (FBS, Gibco, USA) + 1%Antibiotics (Penicillin streptomycin, Gibco, USA). The cell incubator was incubated at 37 °C with 5% CO_2_. Cells in 6-well plates were transiently transfected with small-interfering RNA (siRNA) of CCDC58 and its negative control at 70–80% density using Lipofectamine 3000 (Invitrogen, USA) according to the manufacturer’s description. The siRNA sequences were presented in Table S1. After 48 h transfection, cells were tested for knockdown efficiency of CCDC58 and used for functional experiments.

### RNA isolation and real-time quantitative polymerase chain reaction

Total RNA was isolated with Trizol reagent (Invitrogen) according to the manufacturer’s instructions. The mRNA was reverse transcribed into cDNA using BeyoRT II M-MLV (D7160L, Beyotime). RT-qPCR was performed with 2 × Taq PCR MasterMix (PC1150, Solarbio) and SYBR Green (SY1020, Solarbio) in a Bio-Rad CFX Maestro 2.2 Real-Time PCR System (Bio-Rad, Hercules, CA, USA). β-actin was served as the internal control. The sequences of the primers used were listed in Table S2.

### Western blotting and antibodies

Total cellular protein was extracted using Radio Immunoprecipitation Assay Lysis Buffer (RIPA, Beyotime, China) supplemented with Protease inhibitor cocktail (Beyotime, China) and quantified by Bicinchoninic Acid (BCA kit, Boster, China) assay. Target and reference proteins were separated by 12% sodium dodecyl sulfate–polyacrylamide gel electrophoresis (SDS-PAGE) and transferred to the PVDF membrane (Millipore, USA). Final Western blot visualization was performed using the ECL kit (Beyotime, China). The antibodies involved in this study included anti-CCDC58 (diluted 1:1000, Novus, NBP2-14452, USA) and β-actin (diluted 1:20000, Proteintech, 66009-1-Ig, China).

### Cell counting kit-8 assay and colony formation

Cell counting kit-8 (CCK-8) assay was used to detect cell viability and proliferation. Cells were seeded in 96-well plates at a density of 4000 cells per well and cultured overnight to allow cell adherence. The medium was replaced with fresh medium containing CCK-8 kit (No.C0005, TargetMol). After 4 h of incubation, absorbance was measured at a wavelength of 450 nm using a microplate reader. Cells were seeded into 6-well plates at a density of 1000 cells per well for the colony formation assay. The medium was changed every 3 days, and after 10 days the cells were fixed with 4% paraformaldehyde and stained with 0.5% crystal violet.

### Wound healing and transwell assays

Wound healing assay and transwell assay were used to detect cell migration ability. Cells were seeded in 6-well plates at a density of 50% per well, 2 mL of medium was added and cultured overnight. After siRNA transfection, a straight wound was created in each well using a 200 μL pipette tip. Subsequently, cell debris was washed off with PBS and cultured in DMEM containing 2%FBS. The effect of CCDC58 knockdown on cell migration was analyzed by comparing the wound healing images at 0 and 48 h. Tumor cells were seeded at a density of 5 × 10^4^ cells per well onto the upper chamber (8 μm pore size) containing 200 μL of serum-free medium, while the lower chamber was filled with 500 μL of medium containing 10%FBS. Cells migrated for 24 h. After methanol fixation and crystal violet staining, 5 random fields were selected to collect images, and the number of cells passing through the chamber reflected the migration ability of the cells.

### Statistical analysis

Pearson and Spearman methods were used for correlation analysis. Different methods were selected to calculate the differences between groups according to the data characteristics, including the T-test, Welch t ‘test, Wilcoxon rank sum test, etc. All statistics were performed using R (4.2.1) via R Studio. *P* < 0.05 was defined as statistically significant and all statistical analyses were two-sided tests (*P < 0.05, **P < 0.01, ***P < 0.001, “ns” was regarded as not statistically significant).

## Results

### Expression of CCDC58 in various tumors and normal tissues

**Figure 1:**
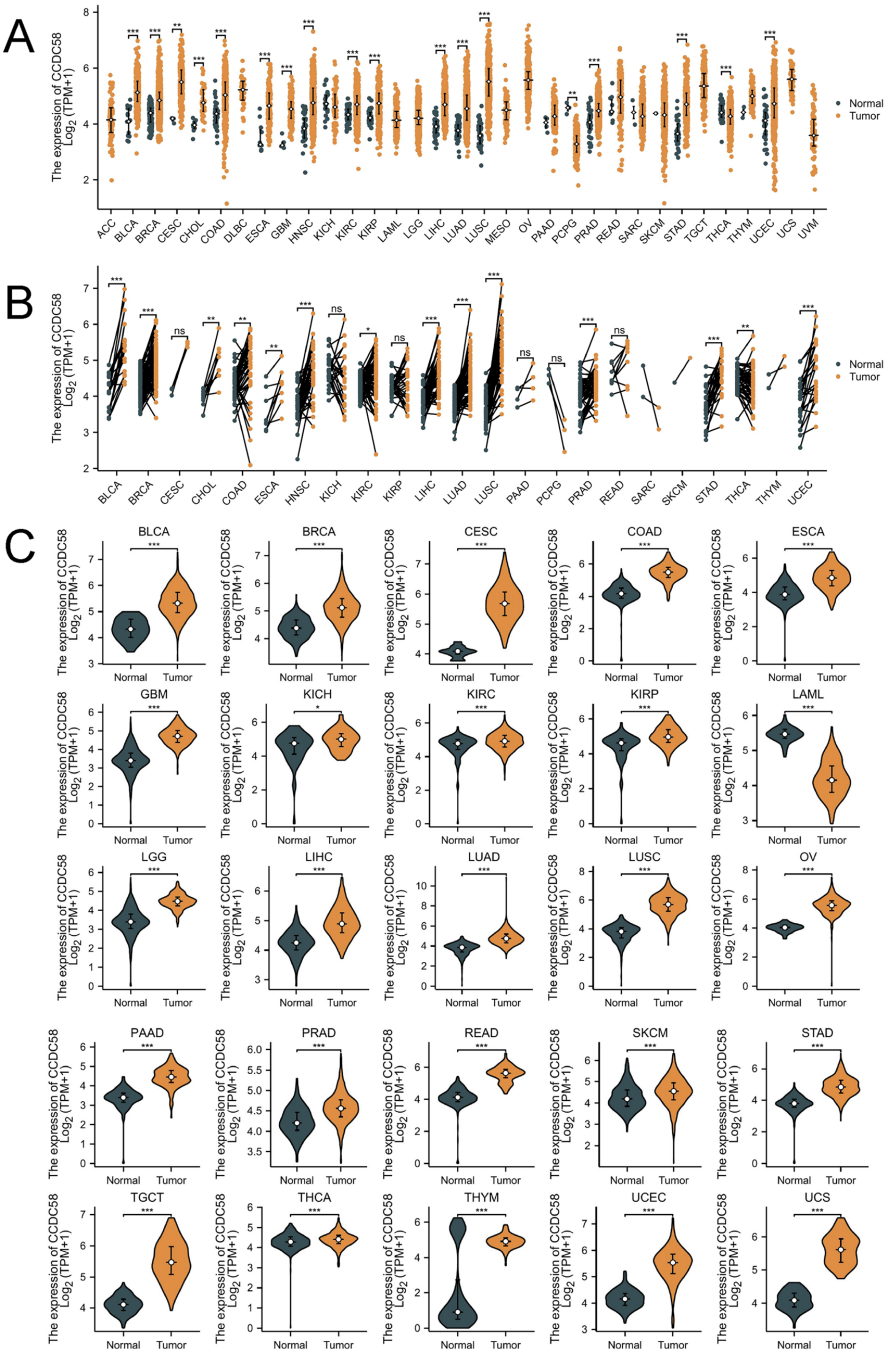
(**A**) The expression of CCDC58 in pan-cancer from TCGA datasets. (**B**) The expression of CCDC58 using paired tumor and normal samples from TCGA datasets. (**C**) The expression of CCDC58 in pan-cancer from TCGA and GTEx.

First, based on TCGA RNA-seq data in STAR workflow, we conducted unpaired samples analysis of mRNA expression of CCDC58 pan-cancer (Fig. 1A). Except for tumors with little or no normal tissue samples, it was not difficult to find that in most cancers, the expression of CCDC58 in tumor tissues was statistically higher than that in normal tissues, including BLCA, BRCA, CESC, CHOL, COAD, ESCA, HNSC, KIRC, KIRP, LIHC, LUAD, LUSC, PRAD, STAD, UCEC. In some cancers, tumor tissue expression was lower, which was statistically significant only in PCPG and THCA. Secondly, based on the TCGA datasets, we extracted and analyzed the tumor and normal samples with corresponding number pairs (Fig. 1B). We observed that out of 23 cancers, 13 tumor tissues expressed higher than their paired normal tissues. This was consistent with the results of unpaired analysis, but there was no longer statistically significant CCDC58 expression in CESC and KIRP. In addition, the results in THCA were also consistent with the unpaired results, which were also lower in tumor tissue expression. Thirdly, we continued to analyze the difference in expression of CCDC58 in pan-cancer after being added to the control of normal tissue samples from GTEx (Fig. 1C). We presented all statistically significant results in the form of violin plots. Most results showed that CCDC58 expression was higher in tumor tissues, except for LAML. Moreover, the expression of CCDC58 in THCA was contrary to the previous result, that the expression in tumor tissue is higher.

**Figure 2:**
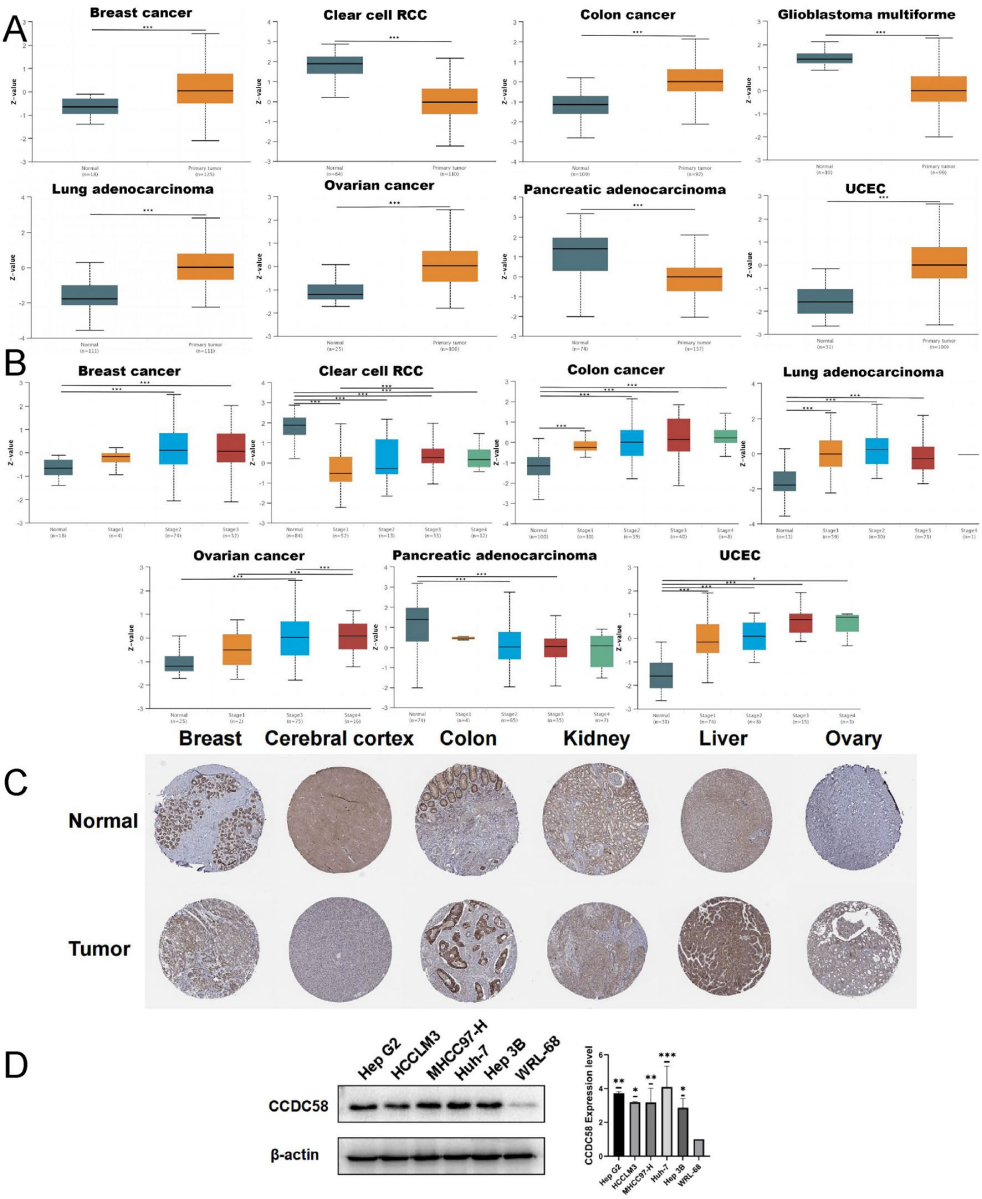
(**A**) The expression of CCDC58 protein in BRCA, Clear cell RCC, COAD, GBM, LUAD, OV, PAAD and UCEC from CPTAC. (**B**) Based on individual cancer stage, the expression of CCDC58 protein in BRCA, KIRC, COAD, LUAD, OV, PAAD and UCEC. (**C**) The expression of CCDC58 protein in tumor and normal tissue of breast, cerebral cortex, colon, kidney, liver and ovary from HPA. (**D**) The expression of CCDC58 protein in cell lines by western blotting.

Next, we analyzed the protein expression of CCDC58 in cancers from CPTAC (Fig. 2A). In our results, the protein expression of CCDC58 was different between the primary tumors and normal tissues in 8 cancers, including BRCA, KIRC, COAD, GBM, LUAD, OV, PAAD, and UCEC. The expression of the CCDC58 protein was higher in tumor tissues in BRCA, COAD, LUAD, OV, and UCEC, but lower in KIRC, GBM, and PAAD. The correlation between CCDC58 protein expression and tumor stage was also detected in CPTAC (Fig. 2B). CCDC58 protein expression was significantly correlated with individual cancer stages in BRCA, KIRC, COAD, LUAD, OV, PAAD, and UCEC. In addition, immunohistochemical images of CCDC58 in multiple organs were obtained from the HPA database (Fig. 2C). It could be seen from the images that CCDC58 protein expression was higher in the breast, colon, liver, and ovary, excluding the cerebral cortex and kidney. This was consistent with the antibody staining score in HPA, which was based on the combination of staining intensity and staining cell proportion. Western blotting results showed that the expression of CCDC58 in liver cancer cell lines was significantly higher than that in normal liver cell line, which was consistent with the data of various databases (Fig. 2D). The full-length blot image was included in Fig. S1.

### Prognostic and diagnosis value of CCDC58 across cancers

**Figure 3:**
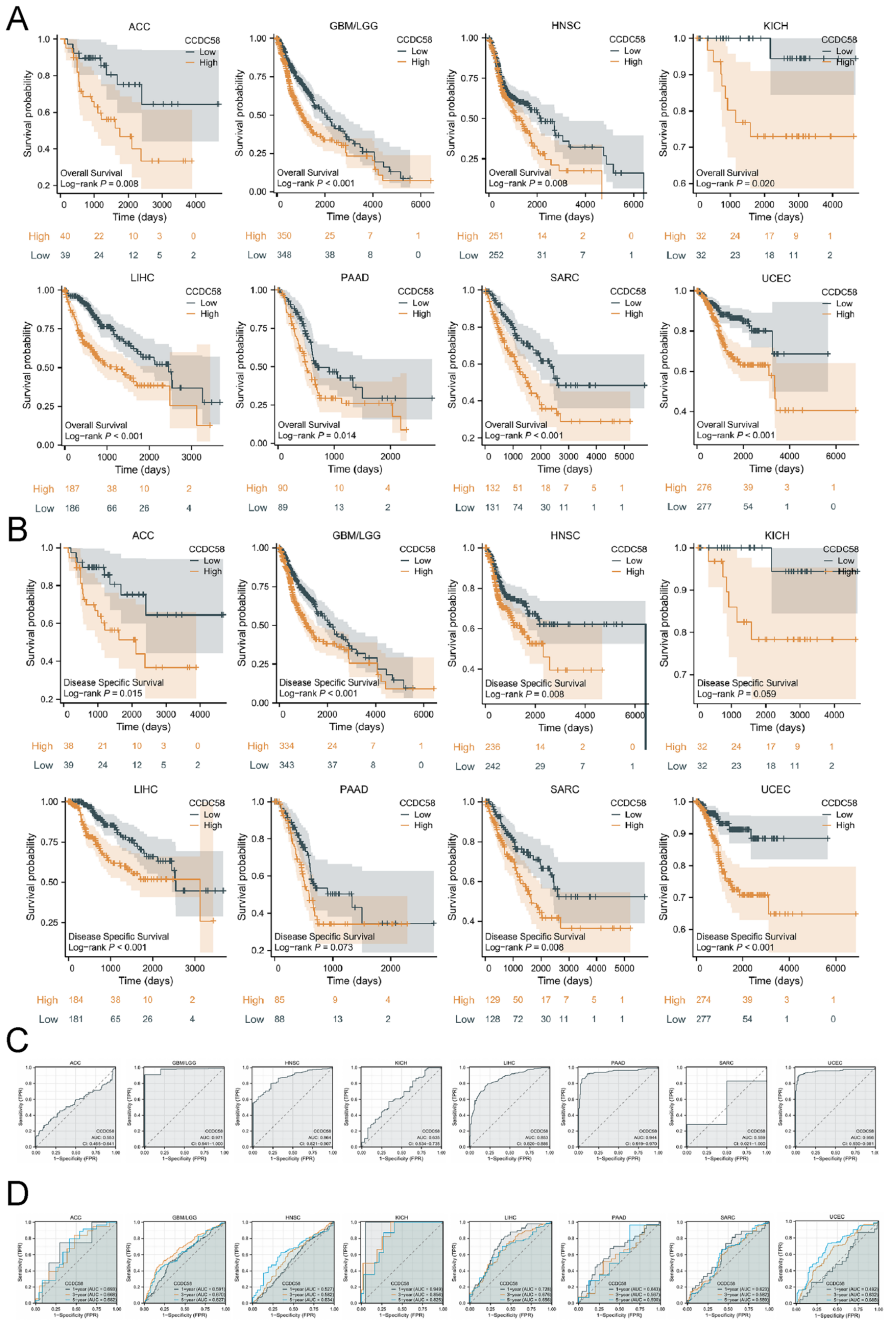
(**A**) OS analysis of CCDC58 genes from TCGA datasets. (**B**) DSS analysis of CCDC58 genes from TCGA datasets. (**C**) ROC curve of CCDC58 genes from TCGA and GTEx(GBM/LGG, HNSC and SARC did not add GTEx). (**D**) Time-dependent ROC curve of CCDC58 genes from TCGA database.

To evaluate the correlation between CCDC58 expression and the prognosis of patients with various tumors, K–M survival curves were plotted, and log-rank tests were performed. We focused on OS and DSS and presented statistically significant results. As shown in Fig. 3A, CCDC58, a high risk factor, linked to poor OS of patients in ACC (*P* = 0.008), GBM/LGG (*P* < 0.001), HNSC (*P* = 0.008), KICH (*P* = 0.020), LIHC (*P* < 0.001), PAAD (*P* = 0.014), SARC (*P* < 0.001) and UCEC (*P* < 0.001). Similarly, in the DSS analysis, CCDC58 was also a high risk factor in ACC (*P* = 0.015), GBM/LGG (*P* < 0.001), HNSC (*P* = 0.008), KICH (*P* = 0.059), LIHC (*P* < 0.001), PAAD (*P* = 0.073), SARC (*P* = 0.008) and UCEC (*P* < 0.001) (Fig. 3B). Detailed statistical results were presented in Tables S3 and S4.

On this basis, we further evaluated the diagnostic value of CCDC58 using ROC curves in the above 8 cancers. The value range of AUC was generally between 0.5 and 1. The closer the AUC was to 1, the better the diagnostic effect of CCDC58 was in predicting the outcome. As shown in Fig. 3C, CCDC58 showed good diagnostic accuracy in GBM/LGG (AUC: 0.971), HNSC (AUC: 0.864), LIHC (AUC: 0.853), PAAD (AUC: 0.944) and UCEC (AUC: 0.956), and average diagnostic accuracy in ACC (AUC: 0.553), KICH (AUC: 0.635) and SARC (AUC: 0.559). In addition, we evaluated the accuracy of CCDC58 in predicting 1-year, 3-year, and 5-year survival using time-dependent ROC curves. In addition, after adding GTEx normal samples, we evaluated the accuracy of CCDC58 in predicting 1-year, 3-year, and 5-year survival using time-dependent ROC curves (Fig. 3D). It showed that CCDC58 predicted survival with better diagnostic accuracy in LIHC (1-year AUC: 0.728, 3-year AUC: 0.676, 5-year AUC: 0.656) and KIHC (1-year AUC: 0.949, 3-year AUC: 0.856, 5-year AUC: 0.825).

### Correlation between CCDC58 expression and critical clinical characteristics

We analyzed the correlation between CCDC58 expression and critical clinical characteristics (Table S5). The expression of CCDC58 in GBM/LGG was correlated with age, WHO grade, and isocitrate dehydrogenase (IDH) status. The higher the expression of CCDC58, the older the patient, the higher the WHO grade, and the lower the mutation rate of IDH. In HNSC, CCDC58 was more highly expressed in male patients, and its high expression was indicative of a higher pathologic stage. And in PAAD, CCDC58 was indicative of a higher pathologic T stage.

### Genetic alteration and DNA methylation of CCDC58

**Figure 4:**
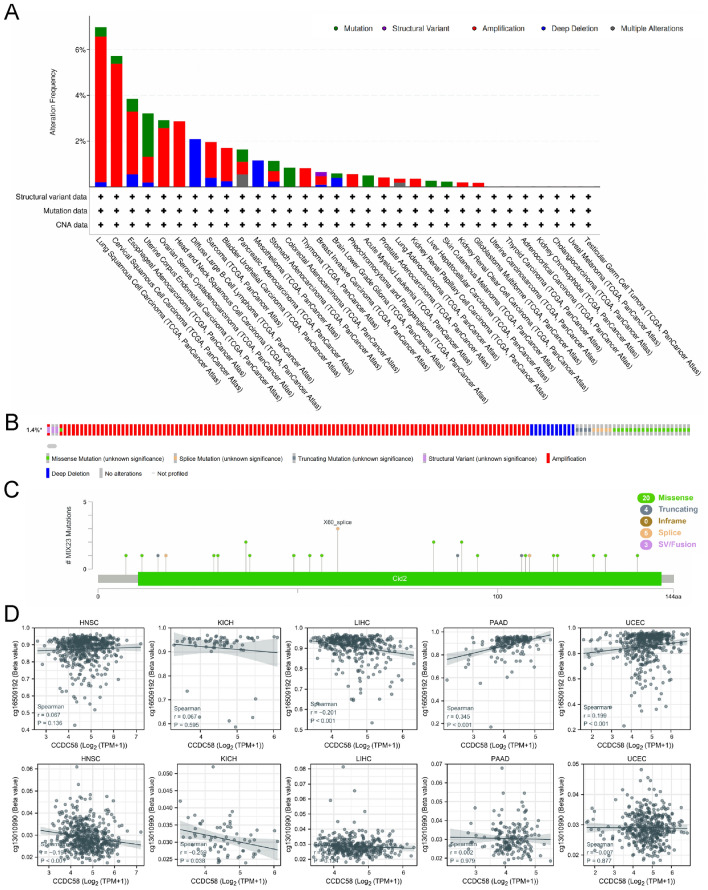
(**A**,**B**) Alteration frequency and alteration types of CCDC58 in cancers. (**C**) Mutation sites and types of CCDC58. (**D**) DNA methylation of CCDC58 in HNSC, KICH, LIHC, PAAD and UCEC.

Detailed gene alteration types and frequency of CCDC58, including mutation, structural variant, amplification, deep deletion, and multiple alterations, were obtained from cBioPortal (Fig. 4A). We observed that CCDC58 amplification accounted for the largest proportion. The top three tumors in alteration frequency were LUSC (mutation 0.41% + amplification 6.37% + deep deletion 0.21%), CESC (mutation 0.34% + amplification 5.39%), and ESCA (mutation 0.55% + amplification 2.75% + deep deletion 0.55%). Figure 4B showed altered/profiled = 156/10950 (1.4%) in CCDC58. In addition, based on cBioPortal, we found that X60_splice was the most important site of the CCDC58 mutation known to date. It was supported by three samples, 2 cases of OV and 1 case of LAML (Fig. 4C).

Through analyzing RNA-seq data from TCGA and Illumina human methylation 450 methylation data using two probes (cg16509192 and cg13010990), we found that CCDC58 expression was negatively correlated with its promoter methylation in HNSC, KICH, and LIHC, positively correlated in PAAD and UCEC (Fig. 4D).

### Genomic heterogeneity of CCDC58

**Figure 5:**
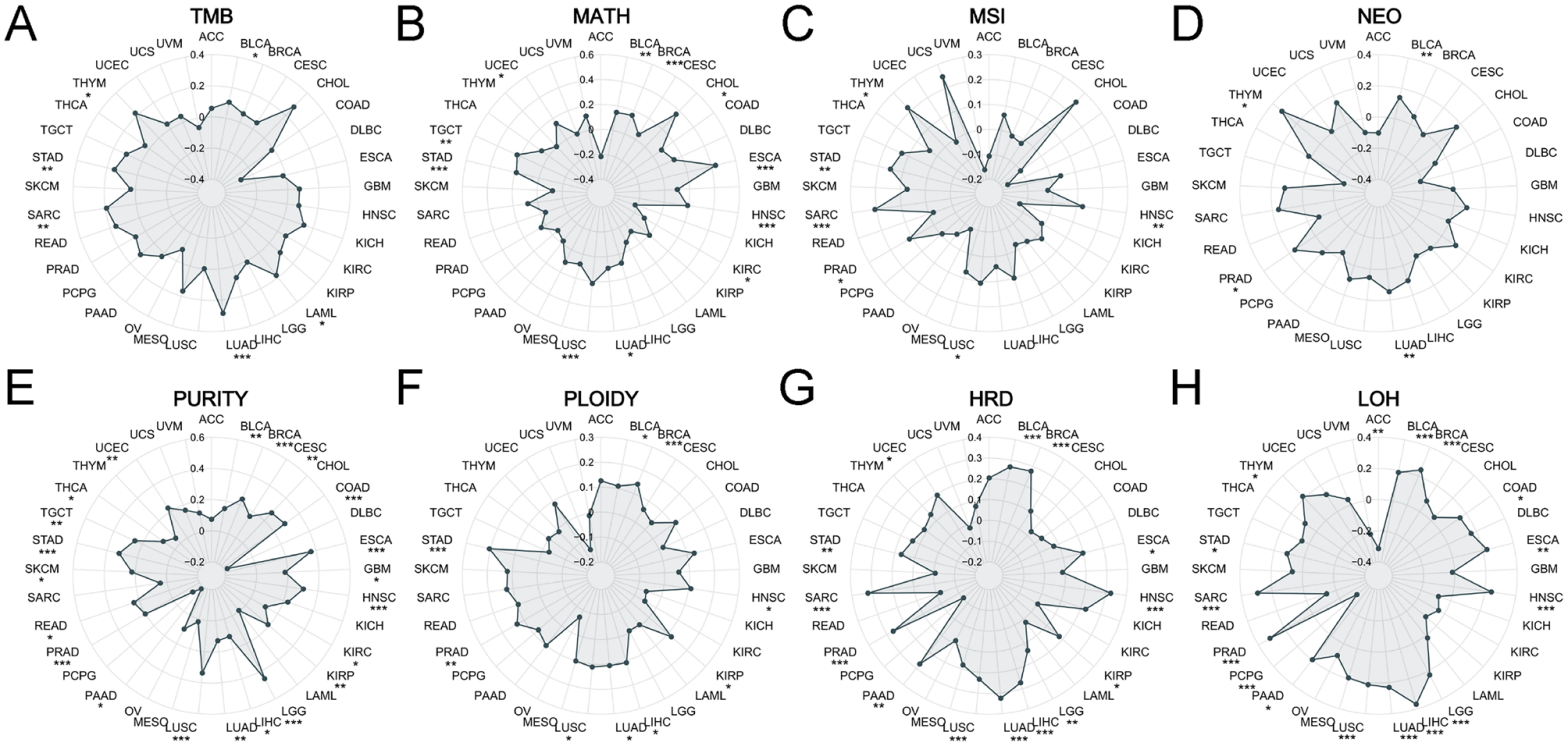
(**A**–**H**) Radar chart of correlation between CCDC58 expression and TMB, MATH, MSI, NEO, PURITY, PLOIDY, HRD and LOH in pan-cancer respectively.

Tumor genomic heterogeneity was highly correlated with the prognosis of tumor immunotherapy. Therefore, we investigated the association of CCDC58 expression with 8 indicators of tumor heterogeneity in pan-cancer, including TMB, MATH, MSI, NEO, PURITY, PLOIDY, HRD, and LOH, to determine if CCDC58 was a predictor of immunotherapeutic responses. CCDC58 expression levels were positively correlated with TMB in BLCA, LAML, LUAD, SARC, STAD, and THYM (Fig. 5A). CCDC58 expression levels were positively correlated with MATH in BLCA, BRCA, CHOL, ESCA, HNSC, LUAD, LUSC, STAD, TGCT, and UCEC, and negatively correlated in KIRC (Fig. 5B). CCDC58 expression levels were positively correlated with MSI in HNSC, LUSC, PRAD, SARC, STAD, and THYM (Fig. 5C). CCDC58 expression levels were positively correlated with NEO in BLCA, LUAD, PRAD, and THYM (Fig. 5D). CCDC58 expression levels were positively correlated with PURITY in BLCA, BRCA, CESC, COAD, ESCA, GBM, HNSC, KIRC, KIRP, LGG, LIHC, LUAD, LUSC, PRAD, READ, SKCM, STAD, TGCT, THCA, and UCEC, negatively correlated in PAAD (Fig. 5E). CCDC58 expression levels were positively correlated with PLOIDY in BLCA, BRCA, HNSC, KIRP, LIHC, LUAD, LUSC, PRAD, and STAD (Fig. 5F). CCDC58 expression levels were positively correlated with HRD in BLCA, BRCA, ESCA, HNSC, KIRP, LGG, LIHC, LUAD, LUSC, PAAD, PRAD, SARC, STAD, and UCEC (Fig. 5G). CCDC58 expression levels were positively correlated with LOH in BLCA, BRCA, COAD, ESCA, HNSC, LGG, LIHC, LUAD, LUSC, PAAD, PRAD, SARC, STAD, and THYM, negatively correlated in ACC and PCPG (Fig. 5H).

### Immune cell infiltration and immune checkpoint genes analysis

**Figure 6:**
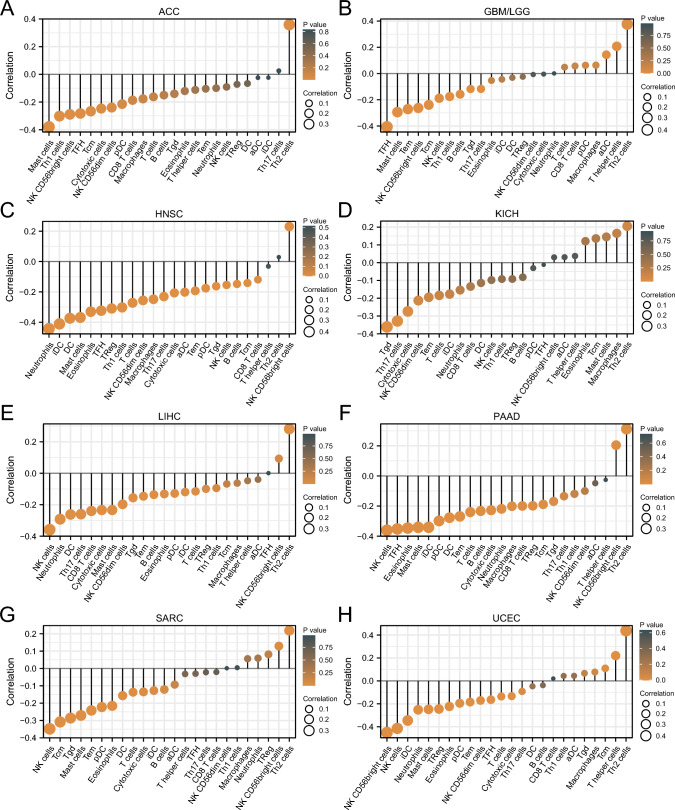
(**A**–**H**) Correlation analysis between CCDC58 and 24 immune cells in ACC, GBM/LGG, HNSC, KICH, LIHC, PAAD, SARC, UCEC.

**Figure 7:**
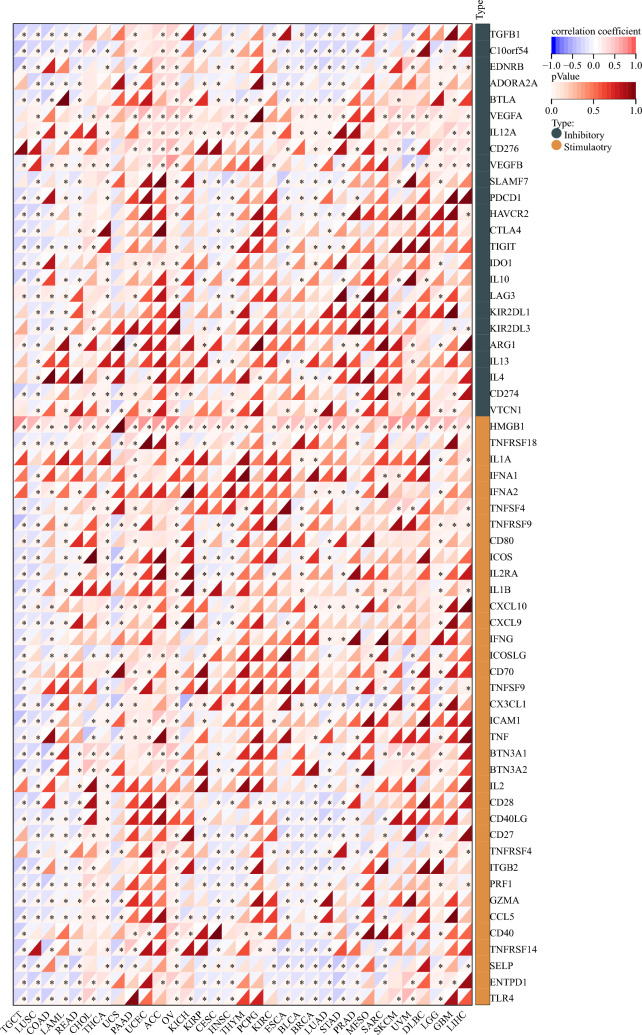
Correlation analysis heatmap of CCDC58 and two types of immune checkpoint marker genes.

In pan-cancer, to determine the value of CCDC58 in influencing the tumor immune microenvironment, we analyzed the correlation between CCDC58 expression and immune cell infiltration in cancers (Fig. 6). The results showed that CCDC58 expression was negatively correlated with most immune cells in ACC, HNSC, LIHC, and PAAD. In GBM/LGG, KICH, SARC, and UCEC, CCDC58 expression was positively correlated with some immune cells. In addition, we established a heatmap of the correlation between CCDC58 and two types of immune checkpoint marker genes. In Fig. 7, we could intuitively see that CCDC58 expression levels were positively correlated with VEGFA, IL12A, CD276, and HMGB1, negatively correlated with C10orf54, EDNRB, CX3CL1, CD27, PRF1, SELP, ENTPD1, and TLR4. In TGCT, LUSC, THCA, OV, CESC, HNSC, ESCA, BLCA, BRCA, LUAD, STAD, and CCDC58 expression was highly correlated with multiple immune checkpoint marker genes.

### Relevance of CCDC58 across functional states in cancers

**Figure 8:**
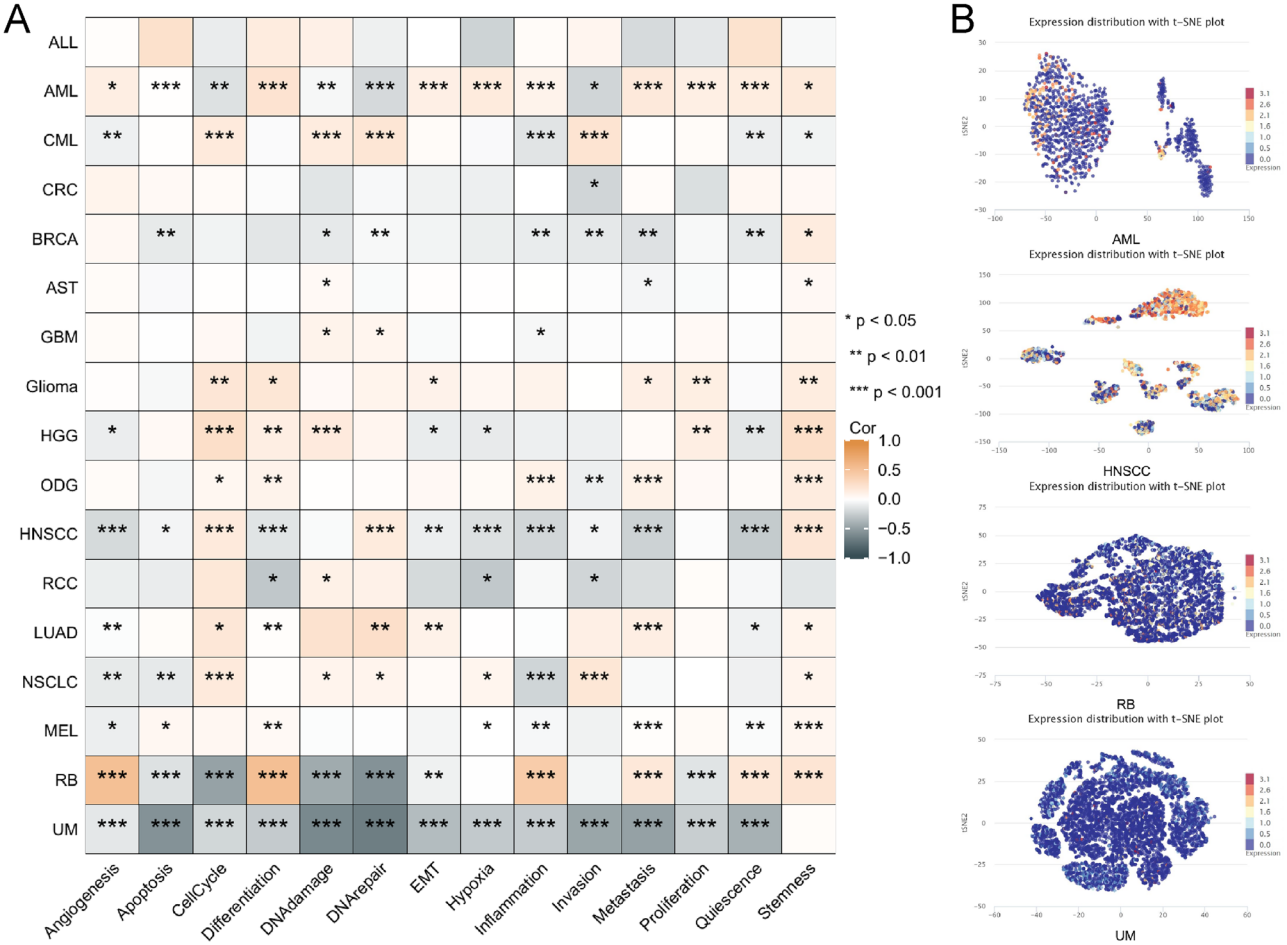
(**A**) Average correlations between CCDC58 and 14 functional states in different cancers. (**B**) CCDC58 expression distribution shown as t-SNE in AML, HNSCC, RB and UM.

Based on single-cell sequencing data from CancerSEA, we established a heat map of the correlation between CCDC58 expression and 14 functional states (Fig. 8A). We observed that CCDC58 expression was highly correlated with these functional states in acute myeloid leukemia (AML), head and neck cancer (HNSCC), retinoblastoma (RB), and uveal melanoma (UM). In AML, almost all of them were positively correlated with CCDC58 expression, except cell cycle, DNA damage, DNA repair, and invasion. In HNSCC, except for cell cycle, DNA repair, and stemness, almost all were negatively correlated with CCDC58 expression. In RB, CCDC58 expression was positively correlated with angiogenesis, differentiation, inflammation, metastasis, quiescence, and stemness, and negatively correlated with apoptosis, cell cycle, DNA damage, DNA repair, epithelial-mesenchymal transition (EMT), and proliferation. In UM, almost all of them were negatively correlated with CCDC58 expression, except stemness. In addition, the expression profiles of CCDC58 at the single-cell levels were presented as a t-SNE diagram (Fig. 8B).

### Results of enrichment analysis of CCDC58 related genes

**Figure 9:**
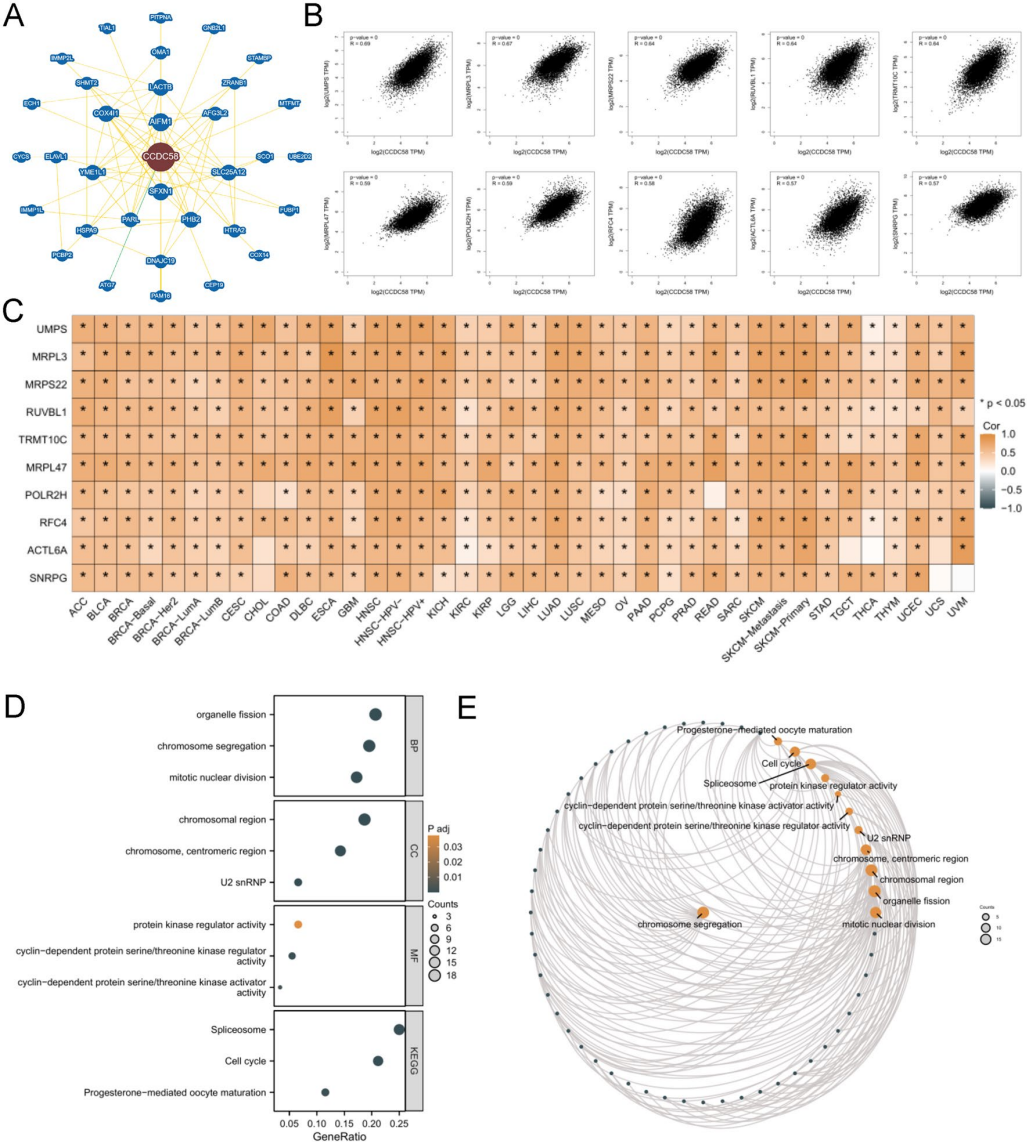
(**A**) Protein interaction network based on CCDC58 from BioGRID (green: association with genetic evidence, yellow: association with physical evidence). (**B**) Scatter plot of correlation analysis of CCDC58 related genes. (**C**) Correlation analysis heatmap of CCDC58 related genes in pan-cancer. (**D**) GO/KEGG enrichment analysis of CCDC58 related genes. (**E**) Enrichment analysis network diagram of CCDC58 related genes.

We performed an enrichment analysis of CCDC58-related genes to explore their potential molecular mechanism in the tumor microenvironment. Firstly, the protein interaction network diagram of CCDC58, including 34 related proteins, was obtained in BioGRID (Fig. 9A). Secondly, the top 100 genes co-expressed with CCDC58 in pan-cancer were obtained in GEPIA2.0 (Table S6). Correlation of UMPS, MRPL3, MRPS22 RUVBL1, TRMT10C, MRPL47, POLR2H, RFC4, ACTL6A, SNRPG with CCDC58 scatter diagram as shown in Fig. 9B. Almost all of these genes showed a high correlation with CCDC58 in pan-cancer. Similar correlations were shown in Fig. 9C from TIMER2.0. Finally, GO/KEGG enrichment analysis was performed for these 10 CCDC58-related genes (Fig. 9D). GO analysis included biological process (BP), cellular component (CC), and molecular function (MF). The BP results showed that CCDC58 was mainly related to organelle fission, chromosome segregation, and mitotic nuclear division. CC of CCDC58 was linked to the chromosomal region, chromosome, centromeric region, and U2 snRNP. In addition, the MF results showed that CCDC58 was also associated with protein kinase regulator activity, cyclin-dependent protein serine/threonine kinase regulator activity, and cyclin-dependent protein serine/threonine kinase activator activity. Finally, the pathways of KEGG enrichment were mainly related to spliceosome, cell cycle, and progesterone-mediated oocyte maturation. Figure 9E showed the enrichment analysis network diagram of CCDC58.

### The expression difference and prognostic value of CCDC58

We further analyzed the differences in CCDC58 expression in different clinical variable subgroups of LIHC and showed the results with statistical differences. Statistical results showed that CCDC58 expression was higher in patients with alpha-fetoprotein (AFP) > 400 ng/ml (Fig. S2A), higher histologic grade (Fig. S2B), worse OS (Fig. S2C) and vascular invasion (Fig. S2E), lower in patients with prothrombin time > 4 s (Fig. S2D) and weight > 70 kg (Fig. S2F). Furthermore, in LIHC, the correlation between CCDC58 expression and molecular and immune subtypes was obtained from TISIDB (Fig. S2G). The results showed that CCDC58 expression was independent of the molecular subtype of LIHC, and was lower in C3 (inflammatory) and higher in C2 (IFN-gamma dominant) and C4 (lymphocyte depleted) in the immune subtype of LIHC.

Subgroup K-M survival curves based on CCDC58 expression levels were plotted to evaluate the role of CCDC58 in predicting survival value in LIHC patients with different variable groups, including age (Fig. S3A), gender (Fig. S3B), body mass index (BMI, Fig. S3C), albumin (Fig. S3D), AFP (Fig. S3E), prothrombin time (Fig. S3F), tumor status (Fig. S3G), pathologic T stage (Fig. S3H), pathologic stage (Fig. S3I). Most of the grouping results were consistent with OS analysis of LIHC, namely, the higher the expression of CCDC58, the worse the prognosis. However, we also found that CCDC58 was of modest value in predicting survival in patients with the female, AFP ≤ 400 ng/ml, prothrombin time ≤ 4 s, pathologic T2 stage, and pathologic stage II.

**Figure 10:**
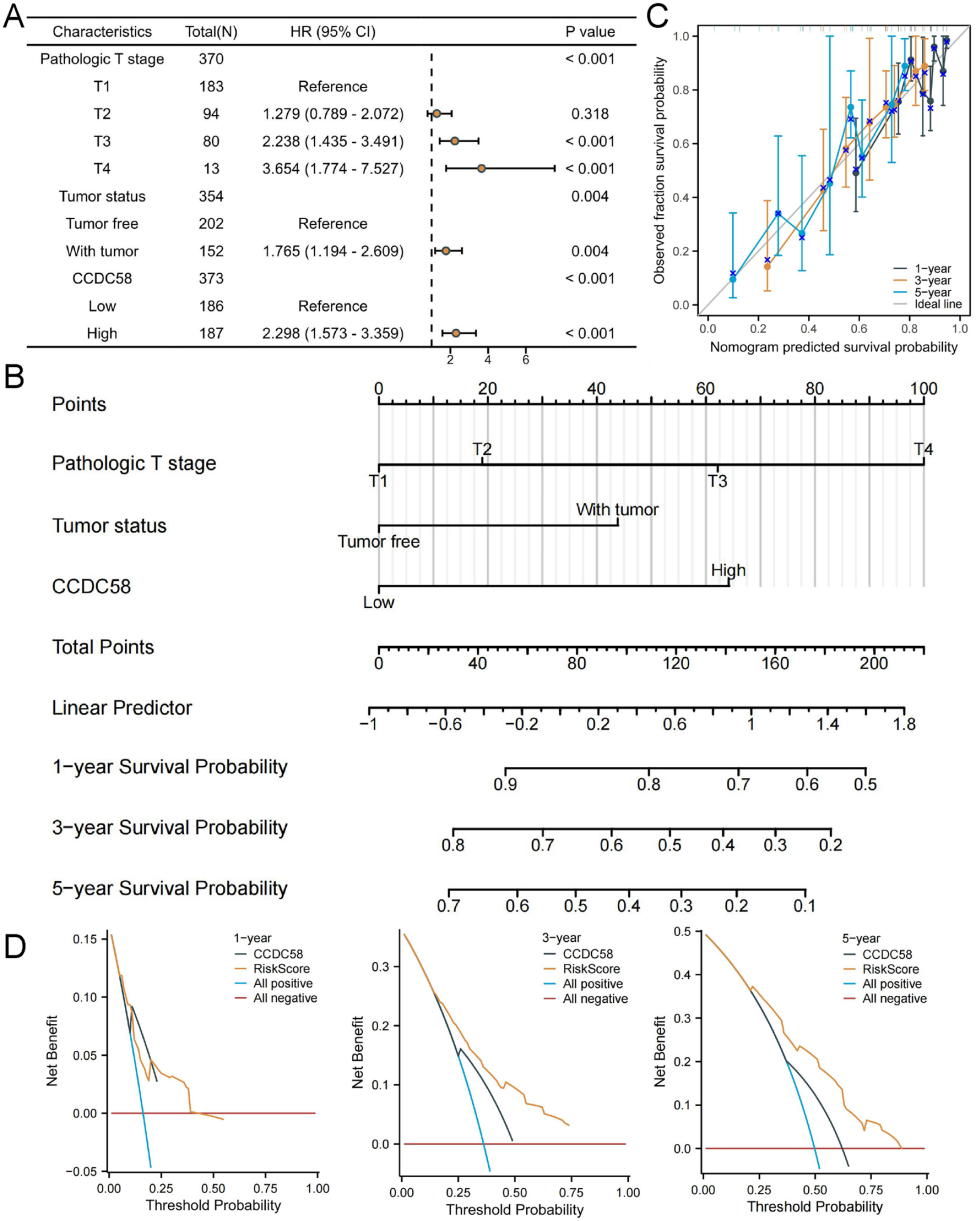
(**A**) Forest plots of multivariate Cox regression results. (**B**) Nomogram model for 1-year, 3-year, 5-year OS of LIHC patients. (**C**) The prognostic calibration curves of nomogram model. (**D**) DCA for 1-year, 3-year, 5-year results.

Cox regression analysis regarding OS was performed using data from LIHC patients in TCGA. Univariate analysis showed that pathologic T stage, Tumor status, and CCDC58 expression were associated with OS. When variables with *P* < 0.1 in univariate results were included, multivariate analysis showed that pathologic T stage, Tumor status, and CCDC58 expression levels were independent risk factors for OS (Table S7). The results of multivariate Cox regression are presented as forest plots (Fig. 10A). The independent risk factors for OS of LIHC patients were drawn into the nomogram model, which could better predict the survival rate of patients at 1-year, 3-year, and 5-year, and the C-index was 0.692 (95%CI: 0.668–0.717) (Fig. 10B). The prognostic calibration result showed that the three fitting curves were well-fitted to the ideal line (Fig. 10C). Finally, DCA was performed to evaluate the clinical utility or patient benefit of the prognostic model, incorporating CCDC58 expression and risk scores generated from Cox regression analysis (Fig. 10D).

Subgroup K-M survival curves of different clinical variables in GBM/LGG, SARC and UCEC also showed an overall trend of poor prognosis with higher CCDC58 expression. However, the predictive value of CCDC58 was different in different intervals of clinical variables. In GBM patients of age ≤ 60 years, high CCDC58 expression pointed to a worse prognosis, while this trend was not evident in patients of age > 60 years (Fig. S4A). Similar patterns were observed for race (Fig. S4C), 1p/19q codeletion (Fig. S4D), IDH status (Fig. S4E), except gender (Fig. S4B). In SARC, the groups in which CCDC58 expression was associated with poor prognosis included: female (Fig. S5B), white (Fig. S5C), leiomyosarcoma (Fig. S5D), non-multifocal tumor (Fig. S5E), R0 residual tumor (Fig. S5F), and not receiving radiotherapy (Fig. S5G). In patients over 60 years old (Fig. S5A), although the statistical significance of log-rank test was not significant, the K-M curve still reflected the trend of high expression of CCDC58 with poor prognosis. In UCEC, patient groups in which CCDC58 expression level was associated with poor prognosis included: BMI ≤ 30 (Fig. S6A), non-diabetes (Fig. S6B), endometrioid (Fig. S6C), post-menopause (Fig. S6D), not treated with radiotherapy (Fig. S6E), white race (Fig. S6F), and weight ≤ 80 (Fig. S6G).

### CCDC58 affected the biological behaviors of LIHC cells in vitro

Few studies investigated the role of CCDC58 in the biological behavior of tumor cells, so we designed siRNA to down-regulate CCDC58 gene. Based on the results of CCDC58 expression in western blot experiments, siRNA was transfected into Huh-7 cells. Since the transfection efficiency of siRNA-3 was less than 70%, siRNA-1 and siRNA-2 were selected for further study (Fig. S7A). CCK-8 assay was used to detect the proliferation of Huh-7 cells. Decreased expression of CCDC58 significantly inhibited the proliferation of Huh-7 cells (Fig. S7B). The number of colonies was reduced in Huh-7 cells after down-regulation of CCDC58 expression (Fig. S7C). Knockdown of CCDC58 also inhibited Huh-7 cells migration in a wound healing assay (Fig. S7D). Transwell assay was used to investigate the migration ability of tumor cells, and CCDC58 knockdown significantly reduced the number of cells passing through the chamber (Fig. S7E). In conclusion, the CCDC58 gene plays an important role in LIHC cell proliferation and migration.

## Discussion

Cancer remained the leading cause of death in all countries in the world^51^. According to statistics, in 2020, there were about 19.3 million new cancer cases and about 10 million cancer deaths worldwide^52^. There were many reasons for the poor prognosis of cancer patients, including tolerance to immunotherapy and differences in the tumor microenvironment^53^. We found that CCDC58 was a powerful pan-cancer biomarker that had rarely been reported. It could be used to assist in the diagnosis and predict the prognosis of patients with a variety of cancers, as well as to evaluate the immune infiltration of tumors. CCDC58 was highly conserved in evolution and a homolog of MIX23. It was widely expressed in various tissues and organs of the human body^54^. At the earliest, CCDC58 was identified in the mitochondrial membrane space as Ybl107c (Mic23) and renamed to avoid confusion with the mitochondrial contact site and cristae organizing system (MICOS)^55,56^. Moreover, other authors had demonstrated that mutations in CCDC58 increased resistance to Ehrlichiae in Drosophila melanogaster^57^.

In the beginning, we investigated the expression of CCDC58 at the transcriptional and translational levels using data from various databases. The results showed that mRNA and protein levels of CCDC58 were significantly upregulated in a variety of tumors compared with adjacent normal tissues. The previous report backed up our results. Some scholars had mentioned that CCDC58 was highly expressed in tumor tissues in triple-negative breast cancer. In addition, MTT and transwell assays showed that overexpression of CCDC58 increased the proliferation, migration, and invasion of tumor cells^13^. The results of western blotting also supported the conclusion of our analysis. It was worth mentioning that the differences in mRNA and protein level results might be due to the metabolism and modification of proteins and different data sources or processing methods in different databases^58^. Second, we evaluated the prognostic and diagnostic value of CCDC58. The expression level of CCDC58 was associated with OS and DSS in 8 cancers, and CCDC58 had good diagnostic efficacy in these cancers, including ACC, GBM/LGG, HNSC, KICH, LIHC, PAAD, SARC, UCEC. Furthermore, CCDC58 had excellent diagnostic performance in GBM/LGG, HNSC, LIHC, PAAD, and UCEC (AUC > 0.85). All in all, the results indicated that CCDC58 had a good predictive ability for the prognosis of patients.

Over the course of a person’s life, spontaneous mutations in somatic cells gradually accumulate. Such mutations can lead to aging and even cancer^59^. Therefore, the Structural variant data, Mutation data, and CNA data of CCDC58 were obtained, and the correlation between CCDC58 and promoter methylation was analyzed. The obtained information showed that in most tumors, the gene alteration types of CCDC58 mainly included mutation, amplification, and deep deletion. DNA methylation is a form of epigenetic regulation. Abnormal DNA methylation includes hypomethylation and hypermethylation, both of which are involved in tumor regulation^60,61^. Our correlation analysis results showed that CCDC58 expression was negatively correlated with DNA methylation in HNSC, KICH, and LIHC, and positively correlated with DNA methylation in PAAD and UCEC. In addition, 8 genomic heterogeneity indicators, including TMB, MATH, MSI, NEO, PURITY, PLOIDY, HRD, and LOH, were also included, which were related to the prognosis or treatment effect of cancer patients. TMB could be used to predict the efficacy of immune checkpoint inhibitors and was a widely recognized predictor^62,63^. Moreover, it has been suggested that MSI status in malignant tumors represents a worse prognosis, such as COAD^64^. Several studies showed that the MATH score could be used to evaluate the effect of patient treatment, such as neoadjuvant chemotherapy for breast cancer patients and EGFR TKI for lung adenocarcinoma patients^65,66^. The results of this study indicated that CCDC58 was associated with multiple tumor heterogeneity indicators and affected the survival and treatment response of patients in a variety of cancers.

The metabolism and secretion of tumor cells can alter the function of the surrounding microenvironment, which is extremely important for tumor progression. Immune cells are an important part of the tumor microenvironment, and the infiltration of immune cells greatly affects the growth and metastasis of tumor cells^67,68^. The results of this study showed that CCDC58 expression was positively correlated with the infiltration of Th2 cells, T helper cells, and NK CD56bright cells in most tumors. NK cells played an important role in monitoring the tumor immune microenvironment and could directly lyse tumor cells without sensitization^69^. NK cells secreted chemokines and cytokines to regulate the immune response, which had great potential in tumor immunotherapy, and the safety of its clinical application had been confirmed^70,71^. It had been well known that Th2 cells mediated immune responses in various allergic diseases, such as asthma^72^. In fact, Th2 cells could initiate an anti-tumor response through the MHC II complex pathway, and their immune response to tumors was largely mediated by the secretion of Th2 cytokines^72,73^. In addition, multiple immune checkpoint genes were also associated with CCDC58 expression. Therefore, we suggested that CCDC58 might affect the sensitivity of cancer patients to immunotherapy by interfering with the tumor immune microenvironment, and it might be a potential therapeutic target and a new biomarker for predicting the prognosis of patients.

The cell cycle was regulated through a complex interaction network, which was closely related to the occurrence and development of tumors^74^. Interference with the regulation of the cell cycle is one of the key mechanisms of continuous tumor cell division^75^. Enrichment results highly suggested that CCDC58 was associated with mitosis, chromosome, cell cycle, and its related proteins. This suggested that CCDC58 might play an important role in the proliferation, apoptosis, and other phenotypes of tumor cells. In addition, we found that CCDC58 was associated with a variety of important clinical variables in LIHC and led to differential prognoses in different subgroups.

Using multiple data sources, this analysis provides a comprehensive picture of the role and underlying mechanism of CCDC58 in pan-cancer, but there are some limitations. First of all, more sequencing results from multi-center clinical samples were needed to improve the reliability of CCDC58 in prognosis and diagnosis. In addition, the regulatory mechanisms of CCDC58 need to be further explored to investigate its effect on tumor progression or immune escape.

### Supplementary Information

Supplementary Information.

## Data Availability

All data involved in this study were obtained from open databases (where specific sources were indicated in the article) or were presented in the Supplementary materials.

## Abbreviations

CCDC: Coiled-coil domain-containing
LIHC: Liver hepatocellular carcinoma
OS: Overall survival
DSS: Disease-specific survival
CI: Confidence intervals
ROC: Receiver operating characteristic
AUC: Area under curve
TMB: Tumor mutation burden
MATH: Mutant-allele tumor heterogeneity
MSI: Microsatellite instability
NEO: Neoantigen
HRD: Homologous recombination deficiency
LOH: Loss of heterozygosity
GO: Gene ontology
KEGG: Kyoto encyclopedia of genes and genome
DCA: Decision curve analysis
