# Supplementary material for: CCDC58 is a potential biomarker for diagnosis, prognosis, immunity, and genomic heterogeneity in pan-cancer
Source: Sci Rep. 2024 Apr 13;14:8575. doi: 10.1038/s41598-024-59154-9 (PMC11014850; doi:10.1038/s41598-024-59154-9)
Supplement: Supplementary file 1 — Supplementary Information. [file 41598_2024_59154_MOESM1_ESM.pdf]

Figure S1

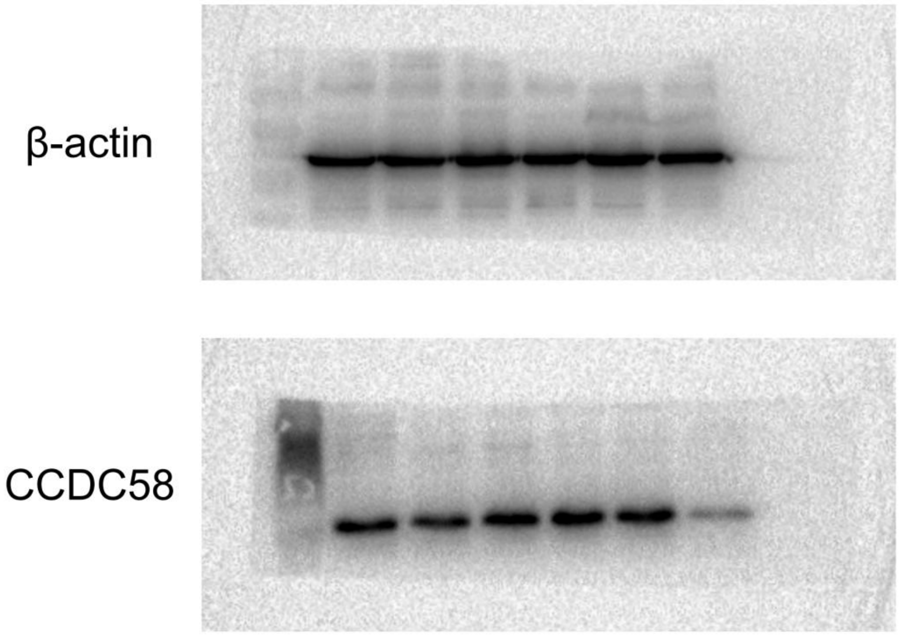

Figure S2

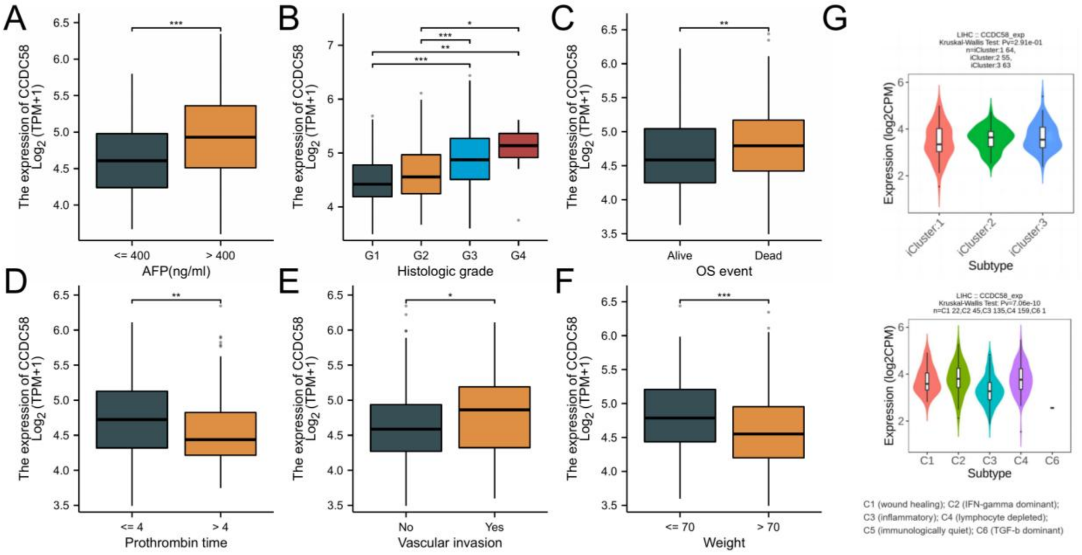
A
B
C
D
E
F
G
The expression of CCDC58 Log2 (TPM+1)
AFP(ng/ml)
<= 400
> 400
Histologic grade
G1
G2
G3
G4
OS event
Alive
Dead
Prothrombin time
<= 4
> 4
Vascular invasion
No
Yes
Weight
<= 70
> 70
LIHC :: CCDC58_exp
Kruskal-Wallis Test: Pv=2.91e-01
n=iCluster:1 64,
iCluster:2 55,
iCluster:3 63
Expression (log2CPM)
iCluster:1
iCluster:2
iCluster:3
Subtype
LIHC :: CCDC58_exp
Kruskal-Wallis Test: Pv=7.06e-10
n=C1 22,C2 45,C3 135,C4 159,C6 1
Expression (log2CPM)
C1
C2
C3
C4
C6
Subtype
C1 (wound healing); C2 (IFN-gamma dominant);
C3 (inflammatory); C4 (lymphocyte depleted);
C5 (immunologically quiet); C6 (TGF-b dominant)

Figure S3

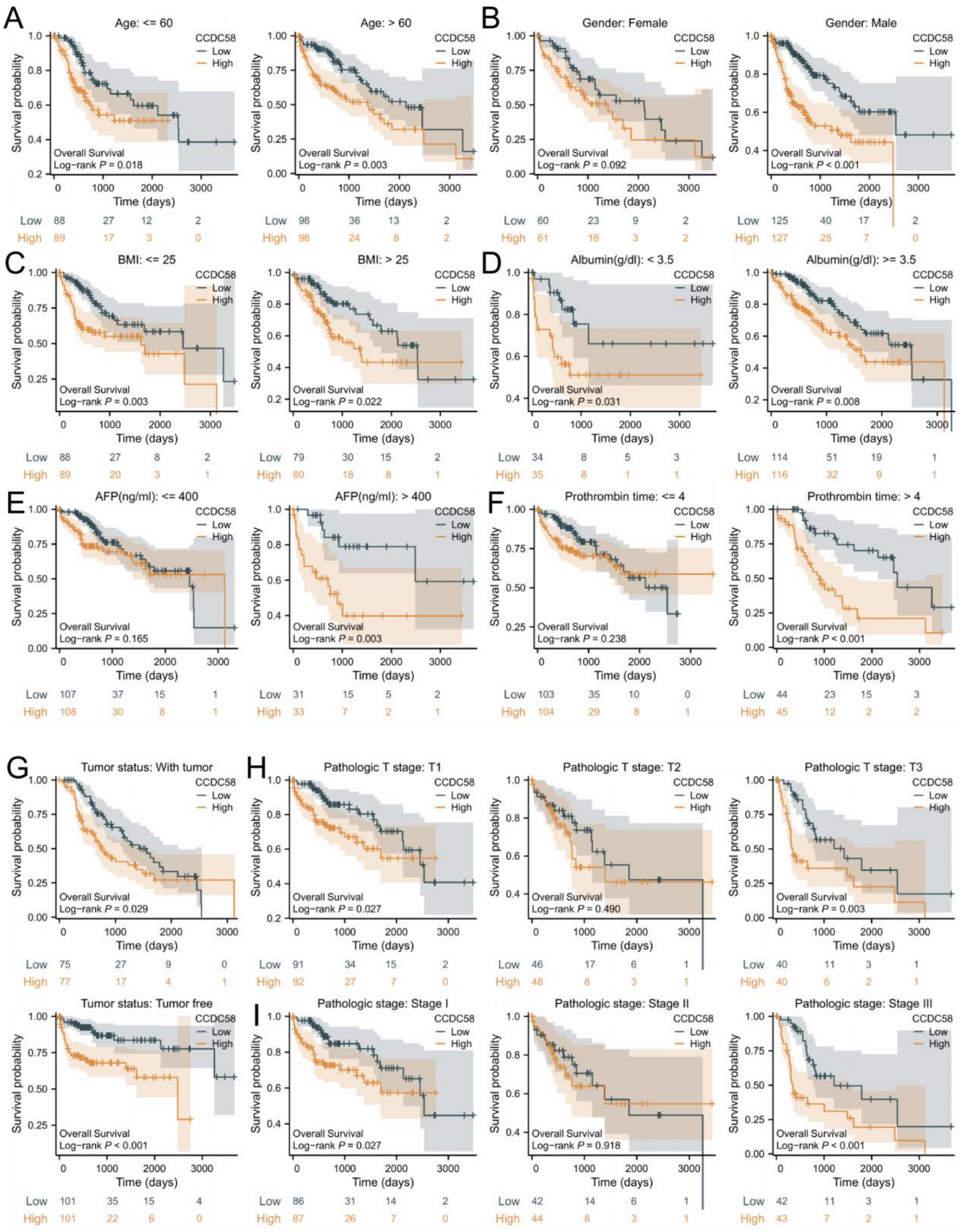

A
Age: <= 60
Log-rank P = 0.018
Age: > 60
Log-rank P = 0.003
B
Gender: Female
Log-rank P = 0.092
Gender: Male
Log-rank P < 0.001
C
BMI: <= 25
Log-rank P = 0.003
BMI: > 25
Log-rank P = 0.022
D
Albumin(g/dl): < 3.5
Log-rank P = 0.031
Albumin(g/dl): >= 3.5
Log-rank P = 0.008
E
AFP(ng/ml): <= 400
Log-rank P = 0.165
AFP(ng/ml): > 400
Log-rank P = 0.003
F
Prothrombin time: <= 4
Log-rank P = 0.238
Prothrombin time: > 4
Log-rank P < 0.001
G
Tumor status: With tumor
Log-rank P = 0.029
Tumor status: Tumor free
Log-rank P < 0.001
H
Pathologic T stage: T1
Log-rank P = 0.027
Pathologic T stage: T2
Log-rank P = 0.490
Pathologic T stage: T3
Log-rank P = 0.003
I
Pathologic stage: Stage I
Log-rank P = 0.027
Pathologic stage: Stage II
Log-rank P = 0.918
Pathologic stage: Stage III
Log-rank P < 0.001
CCDC58
Low
High
Overall Survival
Survival probability
Time (days)

Figure S4

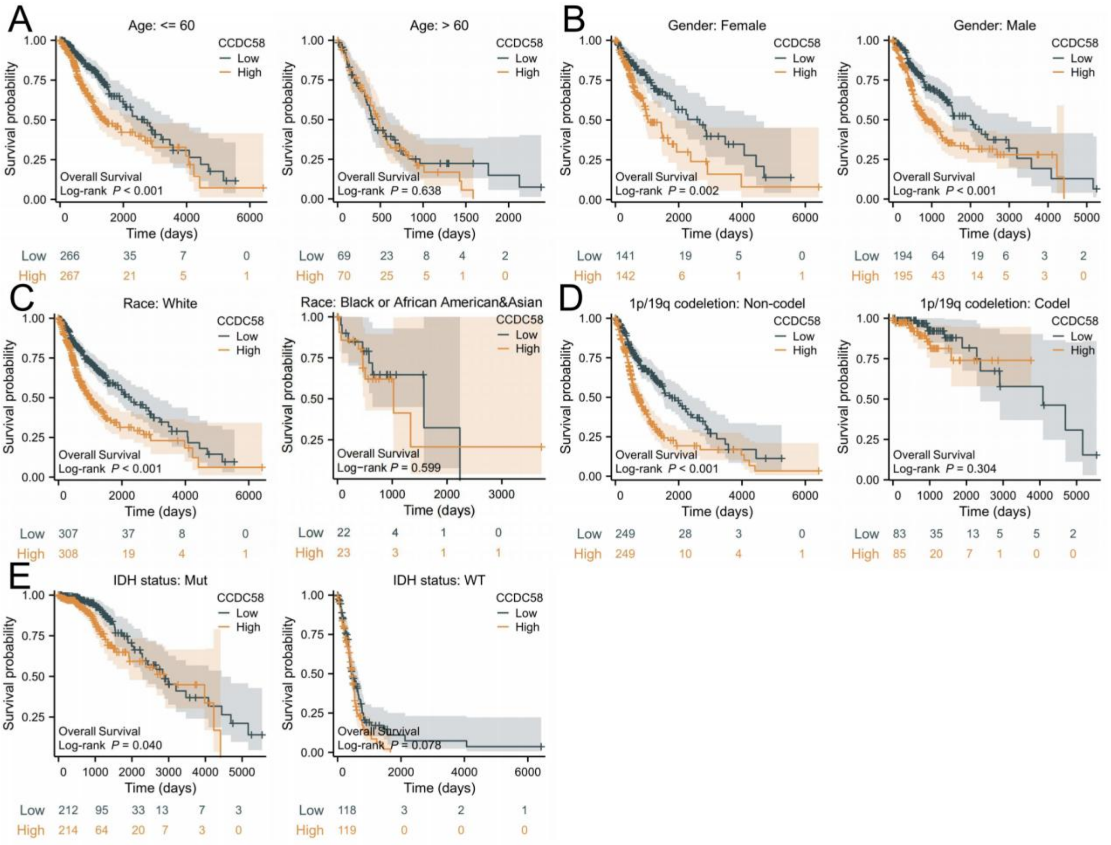
A
Age: <= 60
Overall Survival
Log-rank P < 0.001
Age: > 60
Log-rank P = 0.638
B
Gender: Female
Log-rank P = 0.002
Gender: Male
Log-rank P < 0.001
C
Race: White
Log-rank P < 0.001
Race: Black or African American&Asian
Log-rank P = 0.599
D
1p/19q codeletion: Non-codel
Log-rank P < 0.001
1p/19q codeletion: Codel
Log-rank P = 0.304
E
IDH status: Mut
Log-rank P = 0.040
IDH status: WT
Log-rank P = 0.078
CCDC58
Low
High
Survival probability
Time (days)

Figure S5

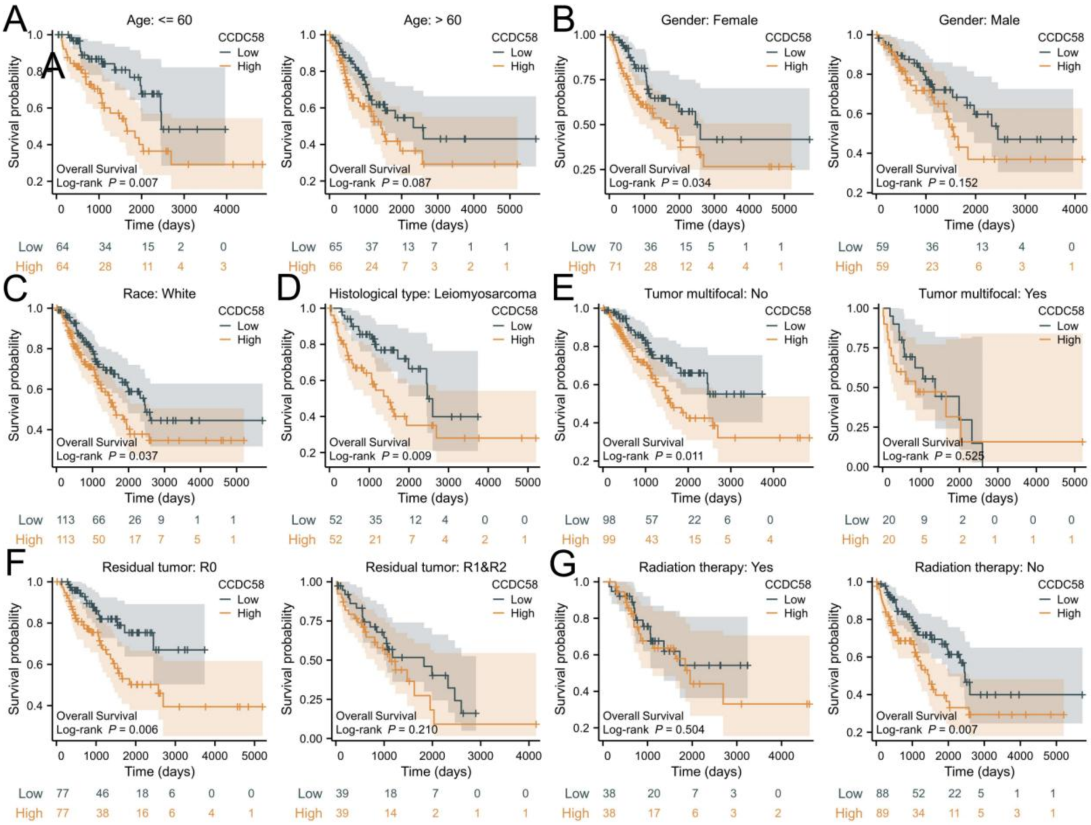
A
Age: <= 60
Age: > 60
B
Gender: Female
Gender: Male
C
Race: White
D
Histological type: Leiomyosarcoma
E
Tumor multifocal: No
Tumor multifocal: Yes
F
Residual tumor: R0
Residual tumor: R1&R2
G
Radiation therapy: Yes
Radiation therapy: No
CCDC58
Low
High
Survival probability
Time (days)
Overall Survival
Log-rank P = 0.007
Log-rank P = 0.087
Log-rank P = 0.034
Log-rank P = 0.152
Log-rank P = 0.037
Log-rank P = 0.009
Log-rank P = 0.011
Log-rank P = 0.525
Log-rank P = 0.006
Log-rank P = 0.210
Log-rank P = 0.504
Log-rank P = 0.007

Figure S6

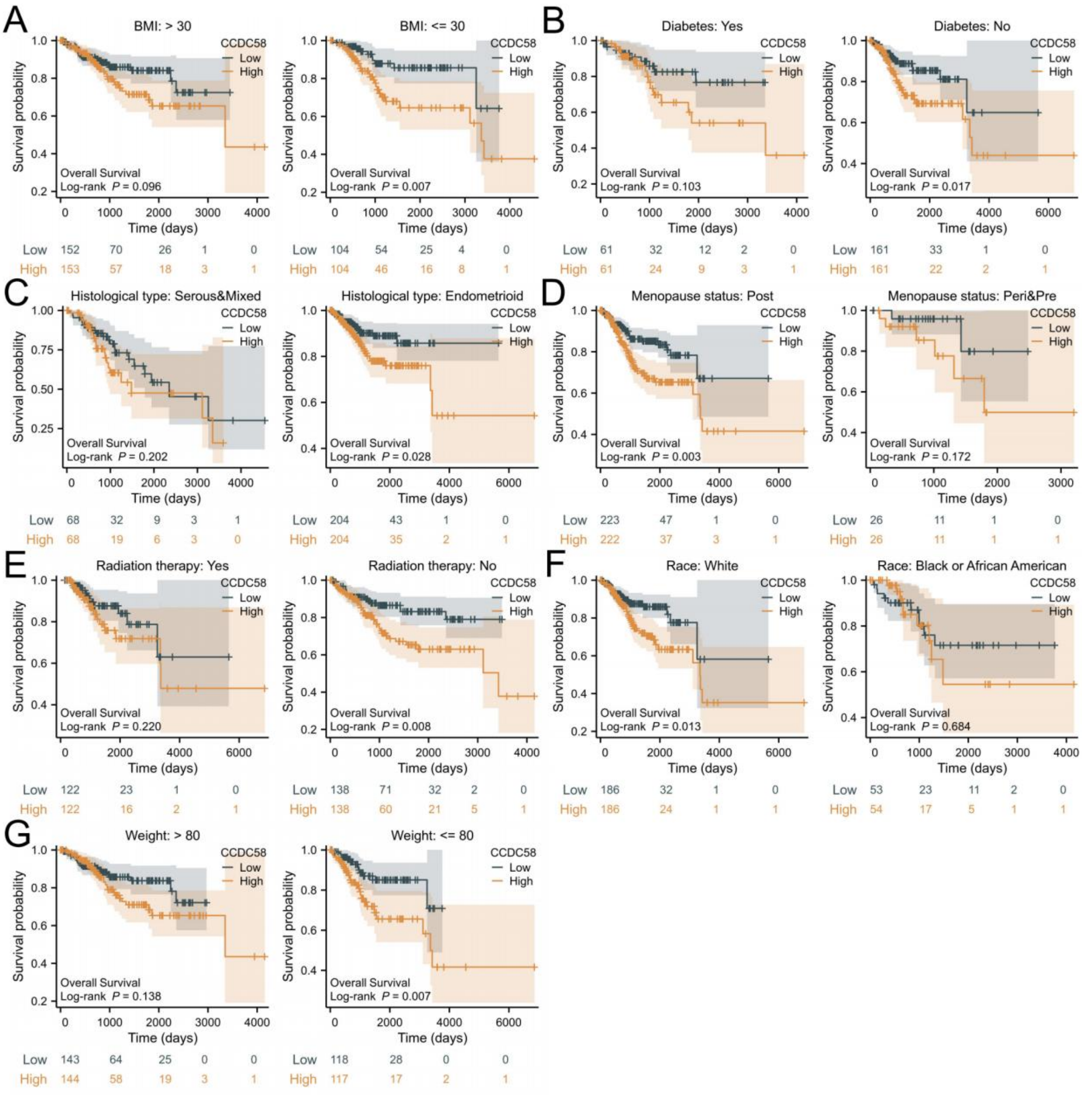
A
BMI: > 30
BMI: <= 30
B
Diabetes: Yes
Diabetes: No
C
Histological type: Serous&Mixed
Histological type: Endometrioid
D
Menopause status: Post
Menopause status: Peri&Pre
E
Radiation therapy: Yes
Radiation therapy: No
F
Race: White
Race: Black or African American
G
Weight: > 80
Weight: <= 80
CCDC58
Low
High
Survival probability
Time (days)
Overall Survival
Log-rank P = 0.096
Log-rank P = 0.007
Log-rank P = 0.103
Log-rank P = 0.017
Log-rank P = 0.202
Log-rank P = 0.028
Log-rank P = 0.003
Log-rank P = 0.172
Log-rank P = 0.220
Log-rank P = 0.008
Log-rank P = 0.013
Log-rank P = 0.684
Log-rank P = 0.138
Log-rank P = 0.007

Figure S7

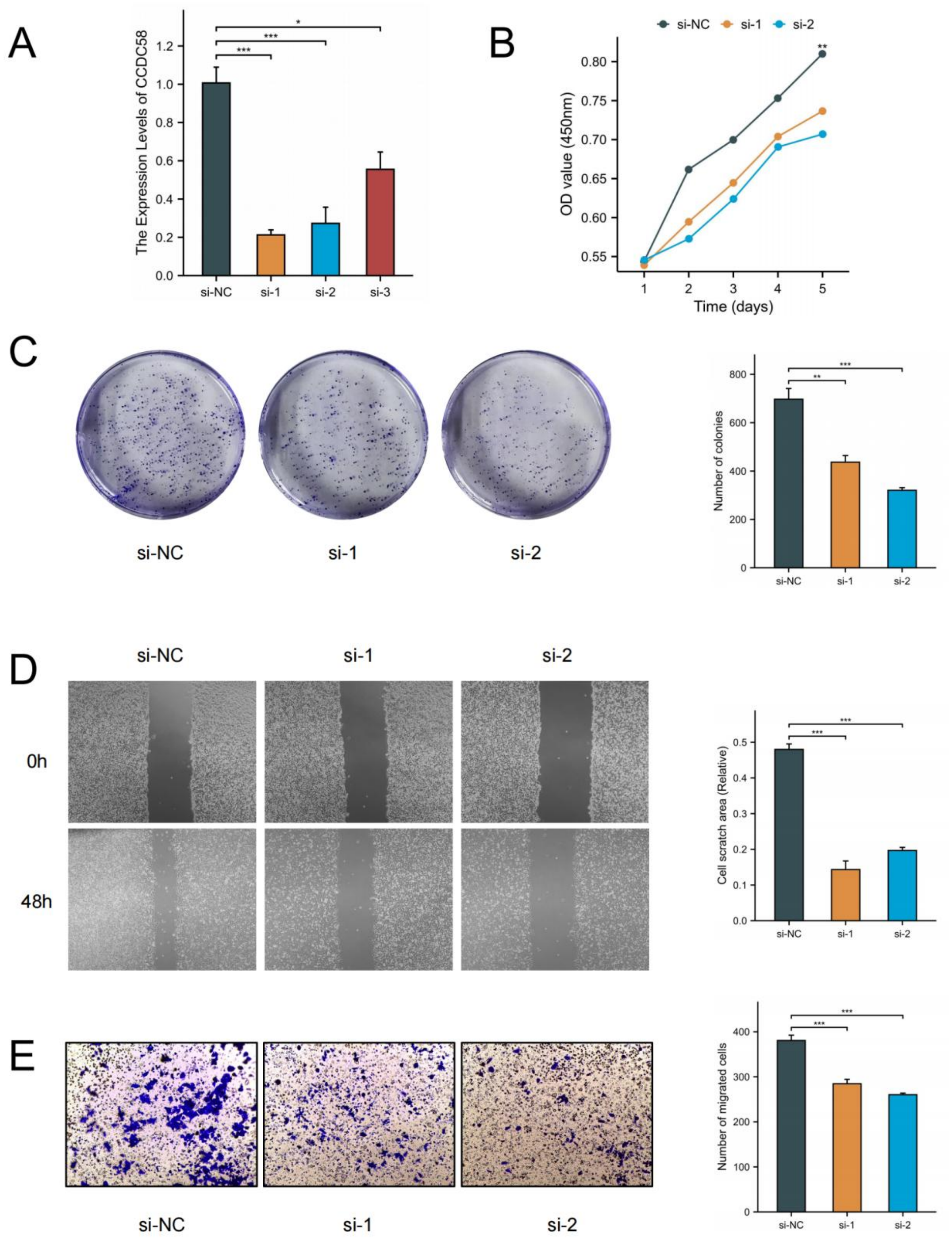

A
The Expression Levels of CCDC58
1.2
1.0
0.8
0.6
0.4
0.2
0.0
*
***
***
si-NC
si-1
si-2
si-3
B
si-NC
si-1
si-2
**
OD value (450nm)
0.80
0.75
0.70
0.65
0.60
0.55
1
2
3
4
5
Time (days)
C
si-NC
si-1
si-2
Number of colonies
800
600
400
200
0
**
***
D
si-NC
si-1
si-2
0h
48h
Cell scratch area (Relative)
0.5
0.4
0.3
0.2
0.1
0.0
***
***
E
si-NC
si-1
si-2
Number of migrated cells
400
300
200
100
0
***
***

Supplementary figure legends:

Figure S1 | The full-length blot image for CCDC58 and β-actin.

Figure S2 | Correlations between CCDC58 expression and grouping variables of LIHC, including **(A)** AFP, **(B)** histologic grade, **(C)** OS event, **(D)** prothrombin time, **(E)** vascular invasion, **(F)** weight. **(G)** Correlations between CCDC58 expression and molecular subtypes and immune subtypes.

Figure S3 | K-M curves between CCDC58 expression and clinical variables of LIHC, including **(A)** age, **(B)** gender, **(C)** BMI, **(D)** albumin, **(E)** AFP, **(F)** prothrombin time, **(G)** tumor status, **(H)** pathologic T stage, **(I)** pathologic stage.

Figure S4 | K-M curves between CCDC58 expression and clinical variables of GBM/LGG, including **(A)** age, **(B)** gender, **(C)** race, **(D)** 1p/19q codeletion, **(E)** IDH status.

Figure S5 | K-M curves between CCDC58 expression and clinical variables of SARC, including **(A)** age, **(B)** gender, **(C)** race, **(D)** histological type, **(E)** tumor multifocal, **(F)** residual tumor, **(G)** radiation therapy.

Figure S6 | K-M curves between CCDC58 expression and clinical variables of UCEC, including **(A)** BMI, **(B)** diabetes, **(C)** histological type, **(D)** menopause status, **(E)** radiation therapy, **(F)** race, **(G)** weight.

Figure S7 | CCDC58 affected the biological behaviors of LIHC cells in vitro. (**A**) The expression of CCDC58 in Huh-7 was detected by RT-qPCR, and siRNA-1 and siRNA-2 were selected for subsequent experiments. CCK-8 assay (**B**) and colony formation assay (**C**) were used to detect the proliferation ability of tumor cells. Wound healing assay (**D**) and transwell assay (**E**) were used to detect the migration ability of tumor cells.

Table S1: The sequences of the siRNAs of CCDC58

siRNA	sequence(5'-3')
siCCDC58-1 S	CAGUAGAGACAGAGUCAUATT
siCCDC58-1 A	UAUGACUCUGUCUCUACUGTT
siCCDC58-2 S	GACGAUUUAACGUUAUUAATT
siCCDC58-2 A	UUAAUAACGUUAAAUCGUCTT
siCCDC58-3 S	GGAUGCAGUCAGAACUGAATT
siCCDC58-3 A	UUCAGUUCUGACUGCAUCCTT
NC CCDC58 S	UUCUCCGAACGUGUCACGUTT
NC CCDC58 A	ACGUGACACGUUCGGAGAATT

S: Sense; A: Anti-Sense

Table S2: Sequences of primers used for amplification of target genes

Gene	Primer sequence (5'-3')
CCDC58 F	GCCCAGACTTCAGCAGTAGTAA
CCDC58 R	GAAGTGAATTCGGCAGCGTT
β-actin F	CTGTGCCCATCTACGAGGGCTAT
β-actin R	TTTGATGTCACGCACGATTTCC

F: Forward; R: Reverse

Table S3: Detailed results of OS prognostic analysis for the eight tumors

OS	Test	Statistical Magnitude	HR	Confidence Interval	*P* value
ACC	Log-rank	6.9732	2.867	1.364 - 6.024	0.0083
GBM/LGG	Log-rank	21.88	1.790	1.397 - 2.293	2.9e-06
HNSC	Log-rank	7.3525	1.435	1.099 - 1.876	0.0077
KICH	Log-rank	5.3942	7.939	2.149 - 29.325	0.0202
LIHC	Log-rank	21.99	2.252	1.587 - 3.196	2.74e-06
PAAD	Log-rank	6.0257	1.675	1.115 - 2.515	0.0141
SARC	Log-rank	11.015	1.951	1.312 - 2.900	0.0009
UCEC	Log-rank	14.512	2.245	1.498 - 3.366	0.0001

Table S4: Detailed results of DSS prognostic analysis for the eight tumors

DSS	Test	Statistical Magnitude	HR	Confidence Interval	*P* value
ACC	Log-rank	5.9435	2.698	1.247 - 5.839	0.0148
GBM/LGG	Log-rank	18.249	1.751	1.346 - 2.277	1.94e-05
HNSC	Log-rank	7.4607	1.614	1.142 - 2.282	0.0076
KICH	Log-rank	3.5748	6.001	1.364 - 26.405	0.0587
LIHC	Log-rank	13.548	2.257	1.437 - 3.544	0.0002
PAAD	Log-rank	3.2225	1.524	0.963 - 2.413	0.0726
SARC	Log-rank	7.0946	1.798	1.162 - 2.781	0.0077
UCEC	Log-rank	17.863	3.109	1.895 - 5.100	2.37e-05

Table S5 | Correlation of CCDC58 Expression and Clinical Characteristics

Characteristics	GBM/LGG			HNSC			PAAD		
	Low expression	High expression	*P* value	Low expression	High expression	*P* value	Low expression	High expression	*P* value
n	349	350		252	252		89	90	
Age, median (IQR)	41 (32, 55)	50 (37, 60)	**< 0.001**	61 (53, 69)	61 (54, 68)	0.970	64.157 ± 10.394	65.067 ± 11.445	0.579
Gender, n (%)			0.974			**< 0.001**			0.946
Female	149 (42.7%)	149 (42.6%)		88 (34.9%)	46 (18.3%)		40 (44.9%)	40 (44.4%)	
Male	200 (57.3%)	201 (57.4%)		164 (65.1%)	206 (81.7%)		49 (55.1%)	50 (55.6%)	
Pathologic T stage, n (%)			/			0.421			**0.013**
T1	/	/		26 (11.6%)	19 (8.5%)		3 (3.4%)	4 (4.5%)	
T2	/	/		72 (32%)	63 (28.3%)		18 (20.5%)	6 (6.7%)	
T3	/	/		43 (19.1%)	53 (23.8%)		64 (72.7%)	79 (88.8%)	
T4	/	/		84 (37.3%)	88 (39.5%)		3 (3.4%)	0 (0%)	
Pathologic N stage, n (%)			/			0.406			0.811
N0	/	/		91 (45%)	80 (38.3%)		24 (27.9%)	26 (29.5%)	
N1	/	/		30 (14.9%)	36 (17.2%)		62 (72.1%)	62 (70.5%)	
N2	/	/		79 (39.1%)	88 (42.1%)		/	/	
N3	/	/		2 (1%)	5 (2.4%)		/	/	
Pathologic M stage, n (%)			/			1.000			0.343
M0	/	/		88 (100%)	100 (99%)		42 (97.7%)	38 (90.5%)	
M1	/	/		0 (0%)	1 (1%)		1 (2.3%)	4 (9.5%)	
Pathologic stage, n (%)			/			**< 0.01**			0.183
Stage I	/	/		20 (9.1%)	5 (2.3%)		11 (12.5%)	10 (11.4%)	
Stage II	/	/		40 (18.2%)	30 (13.9%)		73 (83%)	74 (84.1%)	
Stage III	/	/		35 (15.9%)	44 (20.4%)		3 (3.4%)	0 (0%)	
Stage IV	/	/		125 (56.8%)	137 (63.4%)		1 (1.1%)	4 (4.5%)	
WHO grade, n (%)			**< 0.001**			/			/
G2	140 (43.3%)	84 (26.8%)		/	/		/	/	
G3	132 (40.9%)	113 (36%)		/	/		/	/	
G4	51 (15.8%)	117 (37.2%)		/	/		/	/	
IDH status, n (%)			**< 0.001**			/			/
WT	102 (29.7%)	144 (41.7%)		/	/		/	/	
Mut	242 (70.3%)	201 (58.3%)		/	/		/	/	

Table S6 | Top 100 Genes

Gene Symbol	Gene ID	PCC
UMPS	ENSG00000114491.13	0.69
MRPL3	ENSG00000114686.8	0.67
MRPS22	ENSG00000175110.11	0.64
RUVBL1	ENSG00000175792.11	0.64
TRMT10C	ENSG00000174173.6	0.64
MRPL47	ENSG00000136522.13	0.59
POLR2H	ENSG00000163882.9	0.59
RFC4	ENSG00000163918.10	0.58
ACTL6A	ENSG00000136518.16	0.57
SNRPG	ENSG00000143977.13	0.57
TIMMDC1	ENSG00000113845.9	0.56
CMSS1	ENSG00000184220.10	0.56
FAM162A	ENSG00000114023.15	0.55
RNF7	ENSG00000114125.13	0.55
NDUFB4	ENSG00000065518.7	0.55
CKS1B	ENSG00000173207.12	0.54
MCM2	ENSG00000073111.13	0.53
GMPS	ENSG00000163655.15	0.53
PDCD10	ENSG00000114209.14	0.53
TPRKB	ENSG00000144034.14	0.52
TROAP	ENSG00000135451.12	0.52
CCNB1	ENSG00000134057.14	0.52
UBE2C	ENSG00000175063.16	0.52
WDR53	ENSG00000185798.7	0.52
MAGOH	ENSG00000162385.10	0.52
NUP37	ENSG00000075188.8	0.51
DNAJC19	ENSG00000205981.6	0.51

NCAPH	ENSG00000121152.9	0.51
UBE2T	ENSG00000077152.9	0.51
ISY1	ENSG00000240682.9	0.51
CDK1	ENSG00000170312.15	0.51
RPN1	ENSG00000163902.11	0.51
ECT2	ENSG00000114346.13	0.51
MRPL11	ENSG00000174547.13	0.51
AUNIP	ENSG00000127423.10	0.5
NAA50	ENSG00000121579.12	0.5
CDCA3	ENSG00000111665.11	0.5
MIS18A	ENSG00000159055.3	0.5
TFG	ENSG00000114354.12	0.5
FAM86JP	ENSG00000171084.15	0.5
CCNB2	ENSG00000157456.7	0.5
BRIX1	ENSG00000113460.12	0.5
HMCES	ENSG00000183624.13	0.5
SNRPA1	ENSG00000131876.16	0.49
FAM136A	ENSG00000035141.7	0.49
NDUFB5	ENSG00000136521.12	0.49
GINS1	ENSG00000101003.9	0.49
TOMM5	ENSG00000175768.12	0.49
KIAA1524	ENSG00000163507.13	0.49
RANBP1	ENSG00000099901.16	0.49
C20orf24	ENSG00000101084.16	0.49
MTFR2	ENSG00000146410.11	0.49
CKS2	ENSG00000123975.4	0.49
OIP5	ENSG00000104147.8	0.49
NEK2	ENSG00000117650.12	0.49
GLRX3	ENSG00000108010.11	0.49

BIRC5	ENSG00000089685.14	0.48
METTL5	ENSG00000138382.13	0.48
SNRPB	ENSG00000125835.17	0.48
CENPA	ENSG00000115163.14	0.48
SF3B6	ENSG00000115128.6	0.48
NUF2	ENSG00000143228.12	0.48
CDC20	ENSG00000117399.13	0.48
MAD2L1	ENSG00000164109.13	0.48
NCBP2-AS2	ENSG00000270170.1	0.48
RAN	ENSG00000132341.11	0.48
BOLA3	ENSG00000163170.11	0.48
TTK	ENSG00000112742.9	0.48
ACAD9	ENSG00000177646.17	0.48
SNRPD1	ENSG00000167088.10	0.47
ORC6	ENSG00000091651.8	0.47
GTF2E1	ENSG00000153767.9	0.47
HDGF	ENSG00000143321.18	0.47
RP11-332M2.1	ENSG00000203644.3	0.47
PPIH	ENSG00000171960.10	0.47
VRK1	ENSG00000100749.7	0.47
ZWINT	ENSG00000122952.16	0.47
SEC22A	ENSG00000121542.11	0.47
C17orf53	ENSG00000125319.14	0.47
DPY30	ENSG00000162961.13	0.47
PNO1	ENSG00000115946.7	0.46
LSM5	ENSG00000106355.9	0.46
DPM1	ENSG00000000419.12	0.46
SPC25	ENSG00000152253.8	0.46
CDC45	ENSG00000093009.9	0.46

LSM12	ENSG00000161654.9	0.46
CCNA2	ENSG00000145386.9	0.46
HNRNPA3	ENSG00000170144.18	0.46
TPX2	ENSG00000088325.15	0.46
SRSF7	ENSG00000115875.18	0.46
CDC25C	ENSG00000158402.18	0.46
CEP55	ENSG00000138180.15	0.46
GTPBP8	ENSG00000163607.14	0.46
COA6	ENSG00000168275.14	0.46
TK1	ENSG00000167900.11	0.46
THOC3	ENSG00000051596.9	0.46
PHF5A	ENSG00000100410.7	0.46
ACP1	ENSG00000143727.15	0.46
EXOSC3	ENSG00000107371.12	0.46
CDCA5	ENSG00000146670.9	0.45

Table S7 | Univariate and Multivariate Cox Regression Analysis in LIHC

Characteristics	Total(N)	Univariate analysis		Multivariate analysis	
		Hazard ratio (95% CI)	P value	Hazard ratio (95% CI)	P value
Age	373		0.293		
<= 60	177	Reference			
> 60	196	1.205 (0.850 - 1.708)	0.295		
Gender	373		0.204		
Female	121	Reference			
Male	252	0.793 (0.557 - 1.130)	0.200		
BMI	336		0.234		
<= 25	177	Reference			
> 25	159	0.798 (0.550 - 1.158)	0.235		
Pathologic T stage	370		**< 0.001**		
T1	183	Reference		Reference	
T2	94	1.431 (0.902 - 2.268)	0.128	1.279 (0.789 - 2.072)	0.318
T3	80	2.674 (1.761 - 4.060)	**< 0.001**	2.238 (1.435 - 3.491)	**< 0.001**
T4	13	5.386 (2.690 - 10.784)	**< 0.001**	3.654 (1.774 - 7.527)	**< 0.001**
Tumor status	354		**< 0.001**		
Tumor free	202	Reference		Reference	
With tumor	152	2.317 (1.590 - 3.376)	**< 0.001**	1.765 (1.194 - 2.609)	**0.004**

Characteristics	Total(N)	Univariate analysis		Multivariate analysis	
		Hazard ratio (95% CI)	P value	Hazard ratio (95% CI)	P value
Histologic grade	368		0.792		
G1	55	Reference			
G2	178	1.162 (0.686 - 1.969)	0.576		
G3	123	1.185 (0.683 - 2.057)	0.545		
G4	12	1.681 (0.621 - 4.549)	0.307		
AFP(ng/ml)	279		0.773		
<= 400	215	Reference			
> 400	64	1.075 (0.658 - 1.759)	0.772		
Albumin(g/dl)	299		0.665		
< 3.5	69	Reference			
>= 3.5	230	0.897 (0.549 - 1.464)	0.662		
Prothrombin time	296		0.178		
<= 4	207	Reference			
> 4	89	1.335 (0.881 - 2.023)	0.174		
Fibrosis ishak score	214		0.843		
0	75	Reference			
1/2	31	0.936 (0.437 - 2.002)	0.864		
3/4	28	0.699 (0.288 - 1.695)	0.428		

Characteristics	Total(N)	Univariate analysis		Multivariate analysis	
		Hazard ratio (95% CI)	P value	Hazard ratio (95% CI)	P value
5	9	0.764 (0.181 - 3.228)	0.714		
6	71	0.734 (0.401 - 1.345)	0.317		
Vascular invasion	317		0.169		
No	208	Reference			
Yes	109	1.344 (0.887 - 2.035)	0.163		
Adjacent hepatic tissue inflammation	236		0.771		
None	118	Reference			
Mild	101	1.204 (0.723 - 2.007)	0.476		
Severe	17	1.144 (0.447 - 2.930)	0.779		
CCDC58	373		**< 0.001**		
Low	186	Reference		Reference	
High	187	2.298 (1.610 - 3.280)	**< 0.001**	2.298 (1.573 - 3.359)	**< 0.001**
